# Versatile role of *Pseudomonas fuscovaginae* cyclic lipopeptides in plant and microbial interactions

**DOI:** 10.3389/fpls.2022.1008980

**Published:** 2022-11-08

**Authors:** Enrico Ferrarini, Mihael Špacapan, Van Bach Lam, Andrea McCann, Catherine Cesa-Luna, Bishnu Prasad Marahatta, Edwin De Pauw, René De Mot, Vittorio Venturi, Monica Höfte

**Affiliations:** ^1^ Department of Plants and Crops, Faculty of Bioscience Engineering, Ghent University, Ghent, Belgium; ^2^ International Centre for Genetic Engineering and Biotechnology (ICGEB), Trieste, Italy; ^3^ Department of Chemistry, Faculty of Sciences, University of Liège, Liège, Belgium; ^4^ Centre of Microbial and Plant Genetics, Faculty of Bioscience Engineering, KU Leuven, Leuven, Belgium

**Keywords:** bacterial sheath brown rot, fuscopeptin, syringotoxin, asplenin, cyclic lipopeptides, rice, *Oryza sativa*, *Rhizoctonia solani*

## Abstract

*Pseudomonas fuscovaginae* is the most prominent bacterial sheath rot pathogen, causing sheath brown rot disease in rice. This disease occurs worldwide and it is characterized by typical necrotic lesions on the sheath, as well as a reduction in the number of emitted panicles and filled grains. *P. fuscovaginae* has been shown to produce syringotoxin and fuscopeptin cyclic lipopeptides (CLPs), which have been linked to pathogenicity. In this study, we investigated the role of *P. fuscovaginae* UPB0736 CLPs in plant pathogenicity, antifungal activity and swarming motility. To do so, we sequenced the strain to obtain a single-contig genome and we constructed deletion mutants in the biosynthetic gene clusters responsible for the synthesis of CLPs. We show that UPB0736 produces a third CLP of 13 amino acids, now named asplenin, and we link this CLP with the swarming activity of the strain. We could then show that syringotoxin is particularly active against *Rhizoctonia solani in vitro.* By testing the mutants *in planta* we investigated the role of both fuscopeptin and syringotoxin in causing sheath rot lesions. We proved that the presence of these two CLPs considerably affected the number of emitted panicles, although their number was still significantly affected in the mutants deficient in both fuscopeptin and syringotoxin. These results reveal the importance of CLPs in *P. fuscovaginae* pathogenicity, but also suggest that other pathogenicity factors may be involved.

## Introduction

Sheath rot is an emerging disease that affects rice worldwide. Initially associated with the fungus *Sarocladium oryzae*, it is now recognized that a wide variety of fungal and bacterial pathogens can cause sheath rot symptoms. *Pseudomonas fuscovaginae* is the most prominent bacterial sheath rot pathogen, causing bacterial sheath brown rot (Bigirimana et al., 2015; Quibod et al., 2015). Despite being often described as a disease complex, the individual pathogens rarely occur together: *S. oryzae* is mainly found in lower-altitude fields while *P. fuscovaginae* is typically found at high altitude (Duveiller and Maraite, 1990; Pearce et al., 2001; Musonerimana et al., 2020). *P. fuscovaginae* taxonomically belongs to the *P. asplenii* subgroup within the *P. fluorescens* group (Girard et al., 2021) and causes symptoms that range from necrotic stripes on the sheath to the reduction of panicle emission and grain filling (Zeigler and Alvarez, 1987). Despite being one of the prominent sheath rot causes, *P. fuscovaginae* has received relatively little attention and its pathogenicity and virulence factors are still mostly unknown.

*P. fuscovaginae* produces secondary metabolites called cyclic lipopeptides (CLPs). CLPs are amphiphilic molecules that can interact with and permeabilize biological membranes (Geudens et al., 2017). They are produced *via* complexes of mega-enzymes called nonribosomal peptide synthetases (NRPSs). NRPSs are composed of a sequence of modules, each deputed to the recruitment of a specific amino acid *via* the adenylation domain. For this reason, every NRPS cluster is generally responsible for the production of a specific CLP and its variants (Götze and Stallforth, 2020). NRPSs are encoded by large biosynthetic gene clusters (BGCs) that are normally formed by multiple closely linked genes (Gross and Loper, 2009).

Chemical analysis has revealed that *P. fuscovaginae* UPB264 produces the CLPs syringotoxin and fuscopeptins (A and B) (Ballio et al., 1996; Flamand et al., 1996). Syringotoxin is a member of the “Mycin” family (Girard et al., 2020) and was initially reported in a lemon isolate of *P. syringae* pv. *syringae* ((Gonzalez et al., 1981). It is composed of a 3-hydroxy fatty acid tail of 14 carbons attached to a fully cyclized peptide of nine amino acids. Fuscopeptins belong to the “Peptin” family and have a peptide sequence of 19 amino acids, five of which form a ring, and acylated with a 3-OH-C8 (fuscopeptin A) or 3-OH-C10 (fuscopeptin B) fatty acid. Both compounds play a pivotal role in the pathogenicity of *P. fuscovaginae*. A mixture of pure compounds caused symptoms comparable to the ones caused by the bacteria (Batoko et al., 1997). However, from these results, the effect of the individual CLPs was not clear, and it was not possible to figure out whether the lack of one of these CLPs would affect symptom development.

Both fuscopeptins and syringotoxin show phytotoxic activity. Syringotoxin can form pores in membranes and is toxic to plant tissues (Surico and DeVay, 1982; Serra et al., 1999). Similarly, fuscopeptins are also able to permeabilize membranes (Coraiola et al., 2008). Fuscopeptins’ activity seems to be exerted by inhibition of the plant H^+^-ATPase. Interestingly, syringotoxin alone was not particularly active in inhibiting the H^+^-ATPase but, when combined, a synergistic effect of the two CLPs appeared (Batoko et al., 1998). These results suggest that this synergism could have a pivotal role in the infection process.

Using a random mutation library in *P. fuscovaginae* UPB0736, (Patel et al. (2014) demonstrated that an insertional mutation, in a gene showing high homology to the syringopeptin synthetase C (*sypC*) gene in *P. syringae*, abolished the sheath rot symptoms. Another study showed that the same insertional mutant still considerably affected panicle formation (Peeters et al., 2020). (Weeraratne et al. (2020) identified in *P. fuscovaginae* DAR77795 and DAR77800 homologues of *sypA* of *P. syringae* pv. *syringae*, which takes part in the synthesis of syringopeptin. Site-specific mutation of these genes, in both *P. fuscovaginae* strains, caused a reduction in virulence on rice seedlings and 4-week-old rice plantlets. Although the genes with homology to syringopeptin synthetase genes are likely part of the BGC encoding fuscopeptins, this has not been demonstrated yet.

Recently, a third BGC for a putative lipotridecapeptide (LP-13) was described in *P. fuscovaginae* (Girard et al., 2020). This BGC encodes a CLP, now named asplenin (formerly N5), that was first found in rhizosphere strains from the *P. asplenii* subgroup (Oni et al., 2019; (Oni et al., 2020), in which it may play a role in motility. Several CLPs are deployed by bacteria for swarming motility thanks to their surfactant properties. This type of motility has often been shown in plant-associated bacteria and is involved in root colonization (Raaijmakers et al., 2010).

Next to their phytotoxic activity, fuscopeptins and syringotoxin demonstrated *in vitro* antimicrobial activity. Syringotoxin showed antagonistic activity mainly against yeast strains and at higher concentrations on some filamentous fungi (Sorensen et al., 1996; Girard et al., 2020). Fuscopeptins seem to also have some antimicrobial activity although little has been published on this aspect (Ballio et al., 1996; Girard et al., 2020).

In this study, we investigated the role of CLPs produced by *P. fuscovaginae* UPB0736 in sheath brown rot disease and microbial antagonism. For this purpose, we identified the CLP BGCs in this strain and constructed mutants lacking one, two or all three CLPs by partially deleting the BGC responsible for the production of syringotoxin, fuscopeptins and asplenin. We assessed these mutants for their ability to cause disease and inhibit fungal growth. We show that both fuscopeptins and syringotoxin are involved in sheath rot disease while antifungal activity is mainly due to syringotoxin. Asplenin plays a role in swarming motility but not in pathogenicity.

## Materials and methods

### Biological material and growth conditions

*P. fuscovaginae* UPB0736 was cultured in King’s B medium (20 g/L proteose peptone No.3 (Difco), 1.5 g/L K_2_HPO_4_, 1.5 g/L MgSO_4_.7H_2_O, 10 ml/L glycerol) at 28°C. Solidified medium was supplemented with 15 g/L of bacteriological agar. Liquid cultures were shaken at 150 rpm. Conjugants were selected by supplementing the medium with the appropriate antibiotics: 40 µg/ml gentamycin sulphate (Duchefa Biochemie), 50 µg/ml nitrofurantoin (98%, Alfa Aesar).

*Escherichia coli* strains used are listed in **Table 1**. *E. coli* was cultured in Luria–Bertani medium (10 g/L Tryptone, 10 g/L NaCl, 5 g/L Yeast extract, 15 g/L bacto agar) at 37°C. Liquid cultures were shaken at 150 rpm. Transformants were selected by supplementing the media with the appropriate antibiotics: 15 µg/ml gentamycin, 50 µg/ml kanamycin, 50 µg/ml ampicillin. White-blue screening was performed by adding 80 µg/ml of X-gal (5-bromo-4-chloro-3-indolyl-β-d-galactopyranoside) to the culture medium.

**Table 1:** Microbial strains and plasmids used in this study and their main characteristics.

Strains	Characteristics	Reference
**Bacteria**		
*Pseudomonas fuscovaginae* UPB0736	Wild type (*Oryza sativa*, Madagascar)	(Duveiller and Maraite, 1990)
UPB0736-*Δasp*	Deletion in *aspA* gene	This study
UPB0736-Δ*fst*	Deletion in *fstA* gene	This study
UPB0736-Δ*fus*	Deletion in *fus*A gene	This study
UPB0736-Δ*fst*-Δ*fus*	Deletion in *fstA* and *fusA* genes	This study
UPB0736-Δ*asp*-Δ*fus*	Deletion in *aspA* and *fusA* genes	This study
UPB0736-Δ*asp*-Δ*fst*	Deletion in *aspA* and *fstA* genes	This study
UPB0736-Δ*asp*-Δ*fst*-Δ*fus*	Deletion in *aspA*, *fstA* and *fusA* genes	This study
*E. coli* S17	λpir+, biparental conjugation	(Simon et al., 1983)
*E. coli* DH5α	F-, LacZ Δ M15.	(Hanahan, 1985)
*E. coli* HB101 (pRK2013)	F+, Helper strain for triparental conjugation(Km)	(Ditta et al., 1980)
**Fungi**		
*Rhizoctonia solani* AG 2-1 BK008-2-1	Cauliflower, Belgium	(Pannecoucque et al., 2008)
*Rhizoctonia solani* AG 2-2 CuHav-Rs18	*Phaseolus vulgaris*, Cuba	(Nerey et al., 2010)
*Rhizoctonia solani* AG1-IA SRMX04-3	Cabbage, Vietnam – pathogenic on rice	(Hua et al., 2014)
**Plasmids**		
pEX19gm	Allelic exchange vector (Gm)	(Hoang et al., 1998)
pGEM^®^-T Easy Vector System	Prelinearized vector with 3’-T overhangs	Promega corporation

asp, asplenin, fst, fuscovaginae syringotoxin, fus, fuscopeptin. Bolds are used for grouping.

*Rhizoctonia* strains (**Table 1**) were cultured on Potato dextrose agar (PDA, Becton Dickinson) at 28°C. Dual culture interactions were performed on 1/5 PDA medium (1/5 PDB, Becton Dickinson; 15 g/L bacto agar) at 28°C.

### DNA isolation and genome sequencing

A single colony of *P. fuscovaginae* UPB0736 was inoculated in 5 ml of KB broth and incubated overnight at 28°C, 150 rpm. Two ml of bacteria culture were pelleted (10,000 g, 2 minutes). Genomic DNA was isolated with the Wizard^®^ Genomic DNA Purification Kit (#TM050, Promega corporation, WI, US) following manufacturer instructions. The quality and integrity of the genomic DNA were checked spectrophotometrically (DS-11, DeNovix) and *via* gel electrophoresis. The DNA was quantified with the QuantiFluor^®^ dsDNA system (Promega corporation, WI, US) following the manufacturer’s instructions and measured in a plate reader (Infinite 200 Pro M Plex; Tecan, Switzerland). Genomic DNA fragment size was verified *via* pulsed-field capillary electrophoresis (Femto Pulse System, Agilent, CA, US) with a Genomic DNA 165 kb Kit (Agilent, CA, US). The genomic DNA was sequenced by Eurofins with PacBio (INVIEW *De Novo* Genome 2.0: 5.1 – 10Mb). Assembly and polishing were performed by the company. The single contig genome of *P. fuscovaginae* UPB0736 has been deposited in the NCBI database with accession number CP100603.

### NRPS clusters bioinformatics analysis

The single contig genome of *P. fuscovaginae* was automatically annotated with RAST 2.0 (Overbeek et al., 2013). The annotated genome was then submitted on antiSMASH 5.0 bacterial version (Blin et al., 2019).

Adenylation (A) and condensation (C) domain amino acid sequences from functionally characterized *Pseudomonas* NRPSs were extracted using the PKS/NRPS analysis web-based tool on http://nrps.igs.umaryland.edu/. Amino acid sequences were aligned with MUSCLE 3.8.425 using Geneious Prime (version 11.0.5). A maximum-likelihood tree was constructed with IQ-TREE using the JTT+F+I+G model (Minh et al., 2020). Itol (Interactive Tree Of Life) was used to annotate the tree.

### Mutant construction by two-step allelic exchange

Mutants were generated as described by Hmelo et al. (Hmelo et al., 2015) with some adjustments. The mutants were constructed by deleting about 5000 bp of the first gene of each gene cluster. The gene was blasted on the strain genome with CLC Main Workbench 19.0 (QIAGEN Aarhus A/S) and NCBI BLAST (Johnson et al., 2008). The least conserved region was then targeted for the mutation. Specific primers were designed with Primer-BLAST (Ye et al., 2012) and the appropriate overhangs for the overlap PCR and the restriction sites were included. All primers are listed in **Table S1**.

The two sequences flanking the respective target regions were amplified by PCR and fused by overlap extension PCR following the optimized protocol of Hilgarth and Lanigan (Hilgarth and Lanigan, 2020) with KAPA HiFi HotStart ReadyMix (Roche Diagnostics). The mutant allele was then cloned in the pGEM^®^-T Easy Vector System (Promega Corporation) and transformed into chemically competent *E. coli* DH5α. Transformants were selected for ampicillin resistance, and the correct insertion was screened by white-blue screening and colony PCR. Plasmids were sequence verified by Sanger sequencing. The fused homologous regions were transferred in a pEX19gm allelic exchange vector (Hoang et al., 1998) *via* digestion and ligation using *Xba*I and *Eco*RI-HF restriction enzymes (New England BioLabs inc.). The digested DNA was separated by gel electrophoresis and purified using Wizard^®^ SV Gel and PCR Clean-Up System (Promega corporation). Insert and vector were ligated with T4 DNA Ligase (New England BioLabs inc.) following the manufacturer protocol. The ligation was transformed in *E. coli* S17 or DH5α. Transformants were selected with gentamycin. Final allelic exchange plasmids sequences were verified by colony PCR and sanger sequencing.

One allelic exchange vector was constructed for each NRPS cluster and conjugated in *P. fuscovaginae* UPB0736 wild type (WT) or mutants to obtain single, double and triple mutants for the three CLPs. The mutant allele was transferred by biparental or triparental mating. Donor (*E. coli* DH5a or *E. coli* S17 with pEX19gm+allele), helper (*E. coli* PRK2013-HB101) and acceptor (*P. fuscovaginae* UPB0736*)* were mixed on a KB plate and incubated overnight at 28°C. The merodiploid *P. fuscovaginae* colonies were selected on KB plates with 40 µg/ml gentamycin and 50 µg/ml nitrofurantoin. The isolated merodiploids were counterselected on KB containing 15% sucrose. Markerless mutants and WT colonies were screened by colony PCR and confirmed by Sanger sequencing. The primers used for mutants construction are listed in **Table S1**.

### Mutant characterization by LC-MS/MS

*P. fuscovaginae* WT and mutants were cultured overnight in 5 mL of either KB or LB broth at 28°C (150 rpm). A volume of 10 µl was spotted in duplicate respectively on KB or LB agar plates and incubated for 24 hours. With the help of a sterile cork borer N.1, four agar disks were sampled for each strain from the centre of the two colonies and the area next to them. The four disks were pulled in a single tube and mixed with 500 µl of 70% LC-MS grade Acetonitrile (Biosolve, Valkenswaard, Netherlands).

CLPs extraction was performed by placing the samples in an ultrasonic bath for 10 minutes. Samples were then quickly centrifuged and the supernatant was collected for analysis. Extracted lipopeptides were stored at -20°C before LC-MS/MS analysis.

Ten microliters of extracted lipopeptides were injected and separated on a Waters Acquity UPLC BEH300 C18 column, 15 mm x 1 mm with particle size diameter of 1.7 µm. This separation was achieved on a Waters acquity UPLC I-class instrument. Mobile phase A was composed of 98% MilliQ water and 2% ACN with 0.1% formic acid (FA) (Sigma Aldrich, Overijse, Belgium). Mobile phase B was composed of 98% ACN with 0.1% FA. The initial separation condition consists of 60% mobile phase A. Gradient starts 2 min after injection to reach 0.1% of mobile phase A at 21 min. This is held until 23.5 minutes. At 25 minutes, mobile phase A is set back to 60% for 5 min column equilibration. The total run time is thus 30 minutes. The flow rate was set to 110 µL/min, the column temperature was set to 50°C and the sample temperature was set to 10°C.

MS measurement was performed in positive ionization mode from 100m/z to 2200m/z on a TIMStof instrument (Bruker Daltonics, Bremen, Germany). Dry gas was set to 8 l/min, source temperature was set at 180°C and capillary voltage was set at 3500V. MS/MS analysis was achieved in collision induced dissociation (CID) mode, with a collision energy of 40 eV.

The obtained data were analyzed using Bruker Compass DataAnalysis version 5.3 (Bruker Daltonics, Bremen, Germany).

### Swarming motility assay

Fresh soft LB agar plates were prepared with 6 g/L agar, allowed to dry for 10 min and immediately inoculated. The strains were cultured overnight in 5ml LB broth (28°C, 150rpm). A 5 µl droplet was spotted at the center of the soft LB agar plates and allowed to dry for 20 min. The plates were incubated at 28°C for 24 hours before imaging the plates.

### Antagonistic assays

*P. fuscovaginae* strains were cultured overnight in KB broth at 28°C (150 rpm). A 5 mm PDA plug of a 5 day old culture of *Rhizoctonia solani* AG1-IA strain SRMX04-3, *R. solani* AG 2-1 strain BK008-2-1 and AG 2-2 strain CuHav-Rs18 (**Table 1**) was placed in the center of a 1/5 strength PDA plate with the mycelium in contact with the medium. Ten µl of the *Pseudomonas* strain cultures were spotted on two sides of the plate at 2 cm from the central plug. Each condition was prepared in triplicates. The plates were cultured at 28°C and pictures were taken at 2, 4, and7 days post-inoculation. The mycelial area was measured with Fiji imageJ2 V2.6.0 (Schindelin et al., 2012). Kruskal Wallis tests were performed with the package SciPy 1.7 (Virtanen et al., 2020). Pairwise Dunn tests were performed with the package scikit-posthocs (Terpilowski, 2019).

### Plant bioassays

Rice seeds (*Oryza sativa* cultivar Kitaake) were surface sterilized with 2% chlorine and a drop of Tween20 while shaking for 30 minutes. Seeds were washed 4 times with sterile double distilled water. About 20 surface-sterile seeds were deposited on sterile Petri dishes on top of moistened filter paper. The seeds were pre-germinated in the dark for 5 days. Perforated plastic trays were filled with about 700 g of potting soil. Six seedlings were transplanted into each pot. The plants were then grown in a greenhouse at 26-28°C, with 12h light and 60% relative humidity. The plants were flood irrigated two times a day and fertilized once a week with 250 ml of 10 g/L ammonium sulphate and 20 g/L iron sulphate.

Six-week-old rice plants were used for inoculation as previously described (Rott et al., 1991; Peeters et al., 2020). Briefly, a 24 h culture of *P. fuscovaginae* (KB, 28°C, 150 rpm) was pelleted (3000 g, 2 min) and resuspended in saline solution (0.85% NaCl). A volume of 100 µl of bacterial suspension was loaded in triplicate in a transparent 96 well plate and the absorbance was measured at 600 nm with a plate reader (Infinite 200 Pro M Plex; Tecan, Switzerland). The bacteria were diluted with saline solution until a measured OD600 of 0.1. The bacterial suspension was injected with a 1 ml syringe at 5 cm above the soil until droplets were dripping from the axil of the youngest leaf (approximately 0.5-1 ml). The inoculation was performed in the main tiller. Each treatment consisted of 18 plants distributed in 3 trays. The control plants were injected with saline solution. The plants were incubated for 24 h at 85% RH, 26°C, and 12h light. Then, the plants were transferred to a greenhouse with temperatures ranging from 24 to 28°C, 12h light and 60-70% RH. The experiment was repeated two times: the first experiment was infected about one week before the start of the booting stage, while the second at approximately two weeks before booting.

### Disease scoring and data analysis

Sheath brown rot symptoms were assessed 7 days post-inoculation. The lesion length was measured on the outer side of the infected tiller. The lesion type was assessed with a 0-4 disease index: 0, no symptoms, only injection puncture; 1, brown necrosis around the injection point with grey stripes; 2, light brown to grey symptoms with some small lesions; 3, intense brown necrosis; 4, strong necrosis from dark brown to black and clear lesions on the stem.

Plants were then scored after the grains were filled, approximately 10 weeks after inoculation. Tiller length was measured from the soil surface to the flag leaf collar of the infected panicle. Panicles were placed individually in Petri dishes and dried at 65°C for five days before being weighed. The graphs were made in Python 3.8 (Van Rossum and Drake, 2009) with pandas, matplotlib, seaborn and statannotation (Hunter, 2007; The pandas development team, 2020; Waskom, 2021). Kruskal Wallis tests were performed with the package SciPy 1.7 (Virtanen et al., 2020). Pairwise Dunn tests with Holm-Sidak correction were performed with the package scikit-posthocs (Terpilowski, 2019). *Post-hoc* chi-square tests Benjamini-Hochberg FDR corrected were performed with scipy.stats.chi2_contingency function (Virtanen et al., 2020). The chi-square pairwise comparisons with both values at zero were excluded from the analysis.

## Results

### *P. fuscovaginae* UPB0736 harbors three BGCs for the production of CLPs

**Figure 1:**
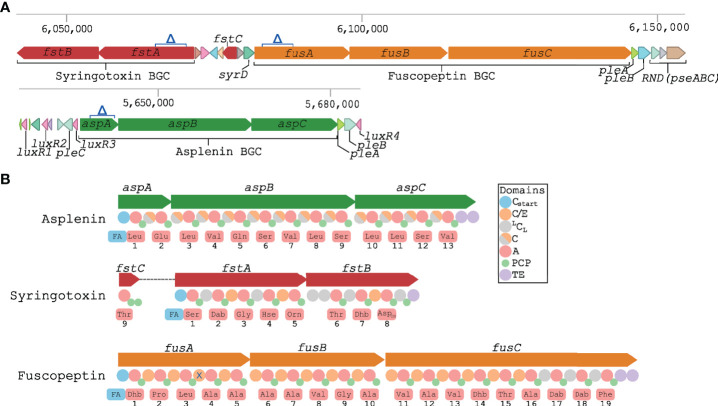
Biosynthetic gene clusters encoding CLPs in *P.* f*uscovaginae* UPB0736. **(A)** Schematic representation of the predicted syringotoxin, fuscopeptin and asplenin NRPS BGC and the associated genes. Δ indicates the deletion in the constructed CLP mutants. Putative transporter genes *pleA, pleB, pleC, syrD, pseABC* and *luxR*-type regulatory genes are also indicated. **(B)** Predicted NRPS domains and adenylation domain amino acid substrate prediction for asplenin, syringotoxin and fuscopeptin synthetases. Predictions were based on phylogeny-based substrate prediction (**Figure S1**–**S3**). A, adenylation: PCP, peptidyl carrier protein; TE, thioesterase. C_start_, lipoinitiation: ^L^C_L_, regular condensation; C/E, condensation/epimerization; C, condensation (epimerization data not available). X: non-functional epimerization.

As previously reported, *P. fuscovaginae* strains can produce the CLPs fuscopeptin, syringotoxin and the recently described asplenin, but the BGCs involved have not been described. PacBio sequencing of strain UBP0736 resulted in a single contig genome that permitted the complete prediction of the three NRPS BGCs. The syringotoxin and fuscopeptin BGCs are closely linked but in reverse strands (**Figure 1A**). There are no regulatory genes linked with these BGCs, but four *luxR*-type regulatory genes (not involved in quorum sensing regulation) are associated with the distantly located asplenin BGC. Putative transporter genes *pleC* (*oprM*-like) and *pleAB* (*macAB*-like) encoding a tripartite export system composed of a cytoplasmic-membrane protein (PleB/MacB), a periplasmic adaptor (PleA/MacA) and an outer membrane protein (PleC/OprM) are located up and downstream of the asplenin BCG (**Figure 1A**, **Table 2**). Putative transporter genes associated with the fuscopeptin/syringotoxin BGC include *syrD*, a second *pleAB* pair, and *pseABC* encoding an RND-type tripartite export system (**Figure 1A**, **Table 2**). In *P. syringae*, the PseABC system is involved in the secretion of both syringomycin and syringopeptin. SyrD is annotated as a cyclic peptide transporter and is in *P. syringae* involved in the transport of syringomycin to the periplasm (Girard et al., 2020).

**Table 2:** CLP biosynthetic gene clusters and associated genes.

Gene	Function	Locus	Protein accession number
**Asplenin BGC**			
*luxR1*	Transcriptional regulator	NLK61_25635	UUQ64548
*luxR2*	Transcriptional regulator	NLK61_25650	UUQ64551
*pleC*	Transporter - OprM subunit - Outer membrane protein	NLK61_25670	UUQ64555
*luxR3*	Transcriptional regulator	NLK61_25675	UUQ64556
*aspA*	NRPS	NLK61_25680	UUQ67959
*aspB*	NRPS	NLK61_25685	UUQ64557
*aspC*	NRPS	NLK61_25690	UUQ64558
*pleA*	transporter - MacA subunit - periplasmic adaptor	NLK61_25695	UUQ64559
*pleB*	transporter - MacB subunit - cytoplasmic membrane protein	NLK61_25700	UUQ64560
*luxR4*	Transcriptional regulator	NLK61_25705	UUQ67960
**Syringotoxin and fuscopeptin BGCs**			
*fstB*	NRPS	NLK61_27150	UUQ64830
*fstA*	NRPS	NLK61_27155	UUQ64831
*fstC*	NRPS	NLK61_27180	UUQ64836
*syrD*	Cyclic peptide transporter	NLK61_27195	UUQ64839
*fusA*	NRPS	NLK61_27200	UXC84427
*fusB*	NRPS	NLK61_27205	UUQ64840
*fusC*	NRPS	NLK61_27210	UUQ64841
*pleA*	Transporter - MacA subunit	NLK61_27215	UUQ64842
*pleB*	Transporter - MacB subunit	NLK61_27220	UUQ64843
*pseA*	RND transporter - outer membrane subunit	NLK61_27225	UUQ64844
*pseB*	RND transporter - periplasmic adaptor subunit	NLK61_27230	UUQ67974
*pseC*	RND transporter - permease subunit	NLK61_27235	UUQ64845

NCBI loci and protein accession numbers.

Bolds are used for grouping.

A phylogeny-based substrate prediction of the fuscopeptin and syringotoxin synthetases was carried out by extracting the adenylation domains of the fuscopeptin and syringotoxin NRPSs and aligning them with adenylation domains of well-characterized mycins and peptins (**Figure S1**). Predictions are indicated in **Figure 1B** and correspond with the chemical structure of the peptide chain of fuscopeptin (19 amino acids) and syringotoxin (9 amino acids). The gene encoding the last adenylation domain of syringotoxin is not part of the NRPS cluster but is located between the syringotoxin and fuscopeptin BGC (**Figures 1A, B**). A similar approach was used to predict the substrate utilization of the adenylation domains of the asplenin NRPS cluster (**Figure S2**). The predicted peptide of asplenin is composed of 13 amino acids (**Figure 1B**). The full structure elucidation of asplenin will be published elsewhere.

Phylogenetic analysis of condensation domains allows to distinguish lipoinitiation (C_start_), regular (^L^C_L_) and co-epimerization (C/E) domains. This enabled a comparison of the inferred peptide stereochemistry with the reported structure of fuscopeptin (fuscopeptin A/B: C8/10-OH Dhb – D-Pro – L-Leu – D-Ala – D-Ala – D-Ala – D-Ala – D-Val – Gly – D-Ala – D-Val – D-Ala – D-Val – Dhb - D-*a*Thr - L-Ala – L-Dab - D-Dab - L-Phe) (Ballio et al., 1996; Baré et al., 1999) and syringotoxin (syringotoxin B: C14-OH L-Ser – D-Dab – Gly – D-Hse – L-Orn – L-*a*Thr – Z-Dhb – L-Asp(OH) – Cl-L-Thr) (Flamand et al., 1996; Bender et al., 1999) (**Figure 1** and **S3**). The bioinformatic prediction matched well with the experimental data, except for the configuration of two fuscopeptin residues, L-Leu3 and D-Dab18. The former indicates that the fourth C/E-type domain of FusA is inactive for epimerization, as observed sporadically in other *Pseudomonas* CLP families (**Figure S3**). In the absence of a separate epimerization domain, the D configuration of the penultimate residue bound to the terminal amino acid (D-Dab18−L-Phe19) by the ^L^C_L_-classified domain of FusC cannot readily be explained. A similar deviation from prediction (D-Dab21−L-Tyr22) is apparent for syringopeptin SP22 (**Figure S3**). However, in the fuscopeptin structural variant jessenipeptin the D-Ser−L-Phe19 dipeptide is consistent with the expected epimerization activity of the terminal C/E-classified domain of the JesC NRPS. With the exception of the eighth C/E domain in JesB lacking epimerization activity, the C domains in the jessenipeptin NRPS system behave as predicted (Arp et al., 2018).

### Partial disruption of NRPS gene clusters inhibits the production of single CLPs

In order to study functional roles of the CLPs in *P. fuscovaginae*, single, double and triple mutants responsible for the synthesis of the three CLPs were constructed by using a two-step allelic exchange resulting in stable markerless deletions as described in the Materials and Methods section. Considering the size of the BGC, it was decided to partially delete the first gene of the cluster (**Figure 1A**). The multi-domain architecture of NRPSs with multiple homologous domain sequences increases the risk of creating off-target mutations. Therefore, the least conserved regions were used in the mutant construction protocol.

LC-MS/MS analysis demonstrated that *P. fuscovaginae* UPB0736 produces fuscopeptin A (**Figure S4**), fuscopeptin B (**Figure S5**), syringotoxin (**Figure S6**) and asplenin (**Figure S7** – predicted structure C10-OH Leu-Glu-Leu-Val-Gln-Ser-Val-Leu-Ser-Leu-Leu-Ser-Val) and that the production of each CLP was abolished in the various mutants (**Table 3**; **Table S2**). In addition, it was evidenced that production was influenced by the growth medium and considerably higher amounts were obtained in KB medium than in LB medium. It was concluded that *P. fuscovaginae* UPB0736 produced the predicted CLP compounds, including fuscopeptin which was originally purified and characterized in *P. fuscovaginae* UPB264 (Ballio et al., 1996) for which no genome sequence is currently available.

**Table 3:** CLP quantification in *Pseudomonas fuscovaginae* UPB0736 WT and CLP mutants by LC-MS/MS.

	Phenotype			LC-MS peak area (% signal to the WT)							
	Fuscopeptins	Syringotoxin	Asplenin	Fuscopeptin A		Fuscopeptin B		Syringotoxin		Asplenin	
Strain				KB	LB	KB	LB	KB	LB	KB	LB
1. UPB0736-WT	**+**	**+**	**+**	100.00	100.00	100.00	100.00	100.00	100.00	100.00	100.00
2. UPB0736-*Δasp*	**+**	**+**	**-**	73.98	59.44	89.25	40.02	70.77	50.13	N/A	N/A
3. UPB0736-Δ*fst*	**+**	**-**	**+**	76.10	31.44	78.91	25.73	N/A	N/A	55.09	34.72
4. UPB0736-Δ*fus*	**-**	**+**	**+**	<0.1	N/A	<0.1	<0.1	17.72	51.11	33.34	121.20
5. UPB0736-Δ*fst*-Δ*fus*	**-**	**-**	**+**	<0.1	N/A	<0.1	<0.1	N/A	N/A	66.88	148.63
6. UPB0736-Δ*asp*-Δ*fus*	**-**	**+**	**-**	<0.1	N/A	<0.1	<0.1	47.63	105.24	N/A	N/A
7. UPB0736-Δ*asp*-Δ*fst*	**+**	**-**	**-**	15.64	11.88	25.23	8.93	N/A	N/A	N/A	N/A
8. UPB0736-Δ*asp*-Δ*fst*-Δ*fus*	**-**	**-**	**-**	<0.1	N/A	<0.1	<0.1	N/A	N/A	N/A	N/A

Samples were taken from KB and LB agar plates. For each CLP, colour filling intensity is proportional to the values. N/A: not available. Orange for + cells in fuscopeptins column, red for + cells in syringotoxin column, and green for + celles in asplenin column.

### Asplenin mutants are unable to swarm

**Figure 2:**
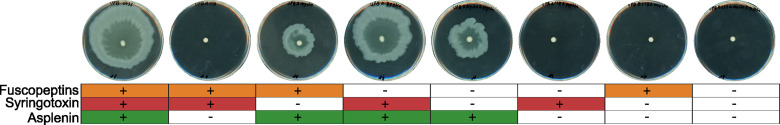
Swarming motility in *P. fuscovaginae* UPB0736 and CLP mutants. Soft agar LB plates were inoculated with an overnight culture of *P. fuscovaginae* UPB0736 or its CLP mutants. The plates were incubated at 28°C and assessed after 24 hours of growth. The experiment was conducted twice, in triplicate, with comparable output. The table below the plates refers to the phenotypes of WT and CLP mutants, as indicated in **Table 3**.

To assess whether any of the three CLPs has a role in the motility of the strain, we tested the knock-out mutants for swarming motility. It was observed that all strains which produced asplenin were able to swarm, however, no swarming occurred in mutants that did not produce asplenin (**Figure 2**). It was also concluded that fuscopeptins and syringotoxin did not play a role in swarming motility under the conditions tested here.

### Syringotoxin is the main compound involved in antifungal activity

**Figure 3:**
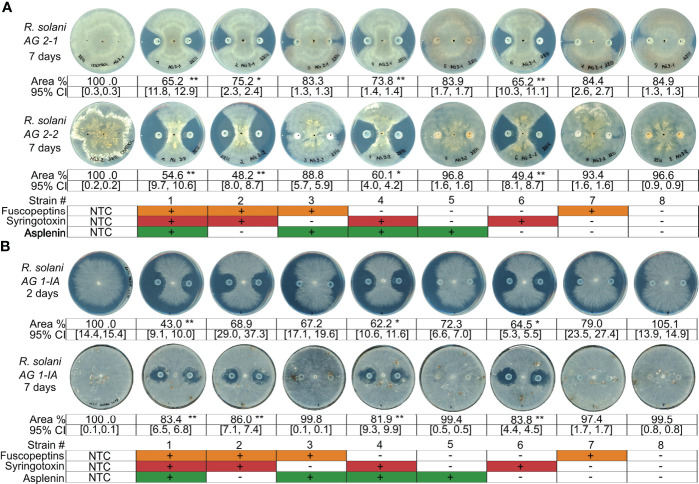
Antagonistic activity of *P. fuscovaginae* UPB0736 and its CLP mutants against *Rhizoctonia solani* strains. **(A)** *Rhizoctonia solani* A2-1 and AG2-2 and challenged with *P. fuscovaginae* UPB0736 and its CLP mutants. Pictures were captured 7 days post inoculation. **(B)** *Rhizoctonia solani* AG 1-IA challenged with *P. fuscovaginae* UPB0736 and its CLP mutants. Pictures were captured 2 and 7 days post inoculation, to take into account the faster growth rate of the strain. Growth rates of the three *R. solani* strains are shown in figure S8. The experiments were conducted at least twice, in triplicate, with comparable output. Values indicate the mycelial area % compared to NTC (full plate). Confidence intervals indicate the 95% confidence interval (normal distribution). The table at the bottom of the figure refers to the phenotypes of WT and CLP mutants, as indicated in **Table 3**. NTC, non-treated control. 95% CI: 95% confidence interval. Statistical significance was calculated with Kruskal-Wallis and *post hoc* pairwise Dunn tests. **: p≤ 1.00e-02, *: 1.00e-02<p≤ 5.00e-02.

*P. fuscovaginae* CLPs have been mainly studied for their phytotoxicity and only preliminary initial data are available about their antifungal activity (Ballio et al., 1996). To further understand the interactions of these CLPs with fungi, we selected *Rhizoctonia solani* AG1-IA (causal agent of rice sheath blight). The *P. fuscovaginae* WT and the mutants that can produce syringotoxin caused a strong and comparable inhibition of fungal growth (**Figure 3A**). However, a small inhibition zone was caused by mutants that produced fuscopeptin but not syringotoxin in the initial time point, for then disappearing. The inhibition zone cause my syringotoxin-producing strains was reduced but still present after 7 days. *R. solani* from the AG 2-1 and AG 2-2 anastomosis groups were also similarly inhibited by the syringotoxin-producing mutants (**Figure 3B**). The inhibition zone was considerably larger than for AG 1-IA. This is most probably due to the considerably faster growth rate of AG1-IA in comparison to the other *R. solani* anastomosis groups (**Figure S8**). Asplenin did not show any antagonistic activity (**Figure 3**). These data suggest a major role of syringotoxin in antifungal activity against *R. solani* but do not exclude that fuscopeptin might play a role in competition with other microorganisms.

### Fuscopeptin and syringotoxin are responsible for the sheath brown rot symptoms

**Figure 4:**
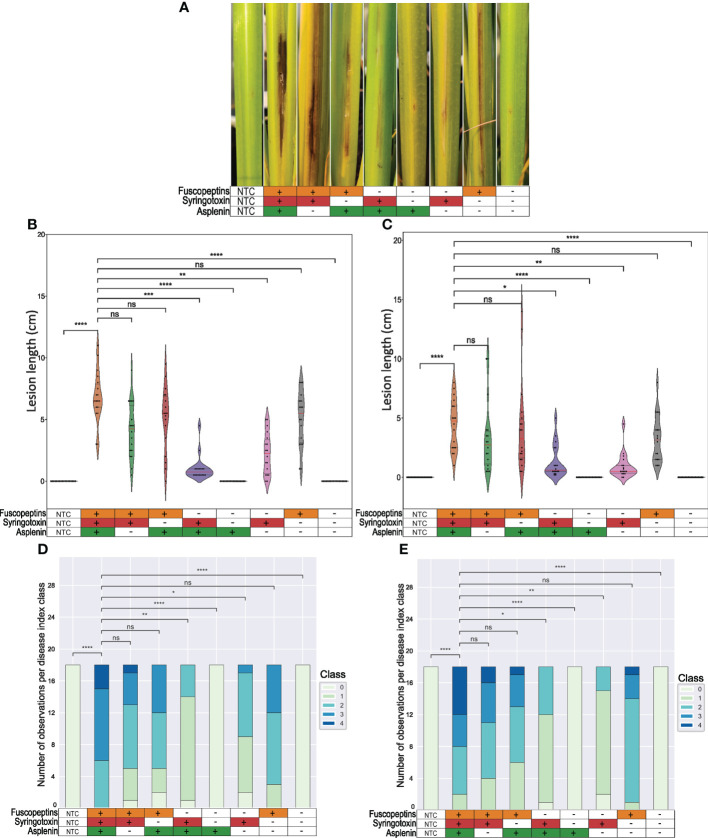
Sheath brown rot symptoms caused by *P. fuscovaginae* UPB0736 and CLP mutants. Rice plants were inoculated by injecting a bacterial suspension of *P. fuscovaginae* UPB0736 WT or mutants for the indicated CLPs. The non-treated controls (NTCs) were injected with saline solution. **(A)** Representative sheath rot symptoms on the infected tiller 7 days post-inoculation (scoring time). **(B, C)** Violin plot showing the length of the necrotic lesions. Dots indicate the measurements for each plant. The red and the two grey lines are respectively the median and the two quartiles. **(D, E)** Stack graphs showing the lesion type based on a 0-4 disease index, in order: no symptoms, only injection puncture; brown necrosis around the injection point with grey stripes; light brown to grey symptoms with some small lesions; intense brown necrosis; strong necrosis from dark brown to black and clear lesions on the stem. Plants were infected about **(B, D)** one week or **(C, E)** two weeks before the start of the booting stage. Tables below the figures refer to the phenotypes of WT and CLP mutants, as indicated in **Table 3**. Statistical significance was calculated with Kruskal-Wallis and *post hoc* pairwise Dunn tests with Holm-Sidak correction. ****, p ≤ 1.00e-04; ***, 1.00e-04 <p≤ 1.00e-03; **, 1.00e-03<p≤ 1.00e-02; *, 1.00e-02<p≤ 5.00e-02; ns, not significant. The pairwise p-values arrays are available in **Tables S5** to **S8**.

To determine the role of the CLPs in causing the sheath rot disease symptoms, rice plant virulence assays were performed (**Figure 4A**). The experiment was performed twice: in the first experiment, the plants were inoculated about one week before the start of the booting stage (**Figures 4B, D**) while in the second experiment the inoculation was performed about two weeks before the booting stage (**Figures 4C, E**). Both experiments showed comparable outputs. The WT strain caused stronger symptoms on plants inoculated closer to the booting stage (**Figures 4B, D**). This can be seen as a further confirmation that the plants become more susceptible at the early booting stage (Bigirimana et al., 2015).

Mutants that could produce fuscopeptin behaved similarly to the WT in causing both lesion length and type, while mutants that did not express fuscopeptin caused significantly milder symptoms. Interestingly, fuscopeptin mutants that produced syringotoxin still caused mild necrosis, while the triple mutant and the one producing only asplenin completely lost the ability to cause necrosis (**Figure 4**). These results indicate that both fuscopeptin and syringotoxin contribute to the typical sheath brown rot symptoms.

### Fuscopeptin and syringotoxin affect panicle formation

**Figure 5:**
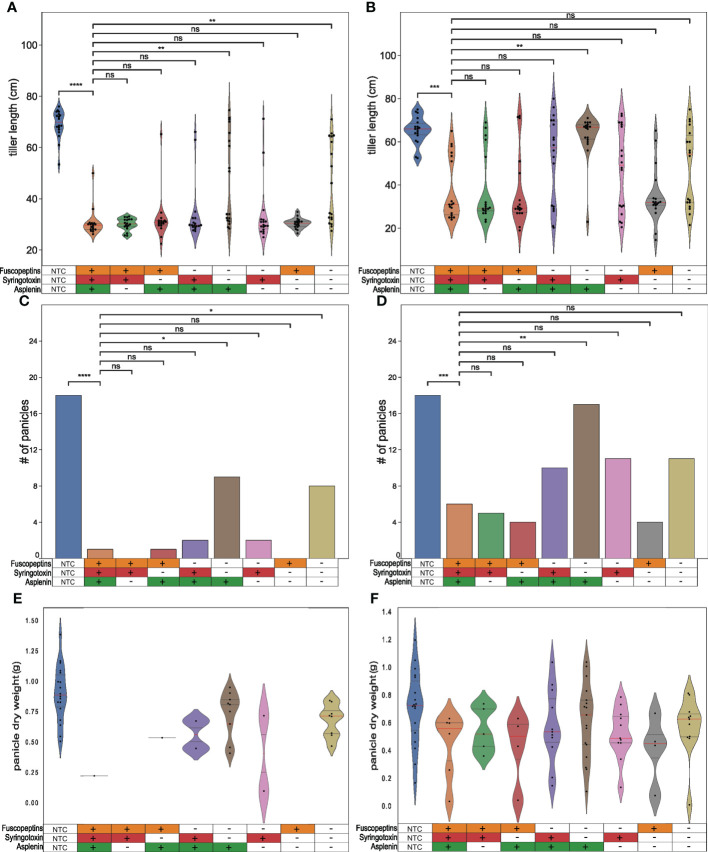
Effect of *P. fuscovaginae* UPB0736 and CLP mutants on tiller length and panicle formation. Rice plants were infected by injecting a bacterial suspension of *P. fuscovaginae* UPB0736 WT or mutants for the indicated CLPs. The non-treated controls (NTCs) were injected with saline solution. Parameters were scored after complete grain filling. **(A, B)** Violin plot showing the tiller length. The dots indicate the single measurements. The red and the two gray lines are respectively the median and the two quartiles. **(C, D)** Number of emitted panicles. **(E, F)** Violin plot showing the panicles dry weight. The dots indicate the single measurements. The red and the two gray lines are respectively the median and the two quartiles. Plants were infected about **(A, C, E)** one week and **(B, D, F)** two weeks before the start of booting stage. Tables below the figures refer to the phenotypes of WT and CLP mutants, as indicated in **Table 3**. **(A, B, E, F)** Statistical significance was calculate with Kruskal-Wallis and *post hoc* pairwise Dunn tests with Holm-Sidak correction. **(C, D)** Statistics was calculated with *post-hoc* chi-square tests Benjamini-Hochberg FDR corrected. ****: p ≤ 1.00e-04; ***: 1.00e-04 <p≤ 1.00e-03, **: 1.00e-03<p≤ 1.00e-02, *: 1.00e-02<p≤ 5.00e-02, ns, not significant. The pairwise p-values arrays are available in **Tables S9** to **S12**.

We previously reported that a *P. fuscovaginae* fuscopeptin mutant affected the panicle formation (Peeters et al., 2020). To determine whether syringotoxin or asplenin can also affect panicle formation, inoculated rice plants were scored after the completion of grain filling for tiller length, the number of emitted panicles and panicle dry weight. It was observed that tiller length was considerably affected in all treatments, except for the non-treated control (NTC) (**Figures 5A, B**). Furthermore, inoculating the plants at an earlier phenological stage resulted in more plants that overcame pathogen infection with a higher number of plants reaching a size comparable to the NTC (**Figure 5B**). The tillers inoculated with mutants that could still produce fuscopeptin and/or syringotoxin were not dissimilar to the WT. However, when both syringotoxin and fuscopeptin production was inactivated, tillers were significantly longer than the ones inoculated with the WT, which also resulted in two different phenotypes with tillers being unable to develop on one side and fully developed on the other (**Figures 5A, B**).

The panicle number (**Figures 5C, D**) and weight (**Figures 5E, F**) were also scored in these *in planta* studies. The number of filled panicles mirrored the results obtained on the panicle length and again this symptom was more severe in plants inoculated close to the booting stage (**Figure 5C**). The effect was most pronounced for fuscopeptin producers but also syringotoxin demonstrated significant phytotoxic activity. When both syringotoxin and fuscopeptin were inactivated, the number of panicles was significantly higher. Asplenin, on the other hand, was not associated with any *in planta* activity.

The strongest effect on panicles dry weight was observed with fuscopeptin-producing strains (**Figures 5E, F**). This effect was however significantly influenced by the lower number of panicles appearing, which might be due to the development of a strong infection upon inoculation.

In summary, this *in planta* data indicates a central role of both fuscopeptin and syringotoxin in pathogenicity but also evidences that other factors apart from CLPs affect panicle formation.

## Discussion

Rice sheath rot disease is an emerging disease that can be caused by several different pathogens. *P. fuscovaginae* is one of the predominant causal agents of the sheath rot disease of rice, often referred to as bacterial sheath brown rot (Bigirimana et al., 2015). This disease received relatively little attention from the scientific community and the role of CLPs and other pathogenicity factors are at large unknown. Most studies focused on sheath rot symptoms and did not investigate further the effect of these toxins on plant yield. To begin to tackle these questions, deletion mutants were constructed in the BGCs of the three CLPs produced by *P. fuscovaginae* UPB0736. A synergistic role of syringotoxin and fuscopeptins was observed in developing sheath rot symptoms but yet other pathogenicity factors are also involved in affecting panicle formation.

It was previously determined that fuscopeptin has strong phytotoxicity on rice (Batoko et al., 1997), but the link between fuscopeptin and disease development has not been addressed. Our *in planta* assay evidenced that fuscopeptin is the main cause of the sheath brown rot symptoms since the *fusA* deletion mutant almost completely lost the capacity to cause necrosis. Fuscopeptin on the other hand did not display significative *in vitro* activity against *Rhizoctonia* strains and it did not play a role in swarming motility indicating that it has a specific function of phytotoxicity. As CLPs often show activity on multiple targets since they can interact with biological membranes (Geudens and Martins, 2018), it cannot be excluded that fuscopeptin could be active against other microorganisms. Therefore, it will be interesting to screen multiple fungi, oomycetes and bacteria to better characterize the range of activity of these CLPs.

Fuscopeptin knockout mutants, that produced syringotoxin, were still able to induce a level of phytotoxicity on the plant sheath. This adds up to previous evidence that pure fuscopeptin and syringotoxin had a synergistic activity *in planta*, although syringotoxin alone did not seem to be active in rice (Batoko et al., 1998). The phytotoxic activity of syringotoxin becomes more evident when considering that the mutants only producing syringotoxin almost completely suppressed the grain production of the infected tiller. This CLP also shows strong *in vitro* antifungal activity against various anastomosis groups of *R. solani*, indicating the multiple roles that syringotoxin plays for *P. fuscovaginae*.

Having a better understanding of the range of antimicrobial activity of fuscopeptin and syringotoxin could open up new agricultural applications. Syringotoxin, in particular, shows strong antifungal activity and has a relatively low phytotoxicity. It could be interesting to also assess whether other crop plants have a different sensitivity to these molecules, especially when applied on the leaf surface.

Unfortunately, the genetic complementation of NRPS BGCs is particularly difficult. The complementation of a *P. fuscovaginae* UPB0736 fuscopeptin mutant was previously attempted, without success (Patel et al., 2014; Weeraratne et al., 2020). This could be due to the large size of the NRPS BGCs, multicopy allele effects of these genes, or polar effects of the mutation on downstream gene products. An alternative approach could be to attempt chemical complementation (D’aes et al., 2014). This requires purified CLPs and therefore it is only possible *in vitro*. For *P. fuscovaginae* UPB0736 this is also hampered by the difficulty of obtaining sufficient amounts of syringotoxin, since it is expressed at very low levels under laboratory conditions.

Our data show that asplenin has no antifungal activity, it does not play a role in the disease severity but is involved in swarming motility. Swarming motility and biofilm formation are traits that are frequently found in plant-associated bacteria and they have been often linked with diverse CLPs (Debois et al., 2008; de Bruijn et al., 2008). These amphiphilic molecules can work as biosurfactants and facilitate bacterial mobility. Therefore, it is reasonable to hypothesize that asplenin could play a role in the motility in or on the plant. The plant inoculation protocol used in the assay can influence the outcome of the result; it is a possibility that the direct injection in the sheath may have masked any motility advantages given by asplenin. Other mycin- and peptin-producing pathogenic *Pseudomonas* strains commonly also produce a third linear lipopeptide of the Factin family with 8 amino acids, such as syringafactin or cichofactin, important for swarming motility (Girard et al., 2020); asplenin seems to play a similar role in *P. fuscovaginae.*

To date, it has been reported that *P. fuscovaginae* can spread *via* seeds and that it can have an epiphytic lifestyle (Duveiller et al., 1988; Batoko et al., 1997). Intriguingly, in our plant assays, the bacteria did not spread from the infected tiller; this could be due to the environmental conditions but also suggests likely limitations in the spread of this pathogen. The presence of asplenin might also indicate a preference of the strain for the rhizosphere environment. We previously isolated closely related bacterial strains from the rhizosphere of rice and cocoyam that also produce asplenin; these isolates do not show pathogenicity at the root level (Oni et al., 2019; Lâm Bạch, 2021).

Four non-quorum sensing *luxR*-type regulatory genes are associated with the asplenin BGC, while no regulatory genes genetically associated with syringotoxin and fuscopeptin BGCs could be identified in *P. fuscovaginae* UPB0736. This was previously also observed for the syringotoxin/fuscopeptin BGC of *P. fuscovaginae* LMG 2158^T^. Also in this strain, which is closely related to UPB0736 and whose CLP BGC organization is essentially the same as for UPB0736, genome mining has revealed the presence of four unrelated *luxR* genes associated with a BGC for a third CLP, called LP-13 (now asplenin) (Girard et al., 2020). A phylogeny of non-quorum sensing LuxR family proteins associated with mycin, peptin and factin BGCs is given in Girard et al. (2020) including the LuxR-type proteins linked to LP-13 (now asplenin). One or more of these LuxR proteins are most probably involved in the transcriptional regulation of asplenin. Similarly, other CLPs, but in particular massetolide and viscosin, also involved in swarming motility, are regulated *via* LuxR-type transcriptional regulators (de Bruijn et al., 2008; de Bruijn and Raaijmakers, 2009; Girard et al., 2022). We are currently investigating whether some of the asplenin-associated LuxR regulators also play a role in coordination of syringotoxin and fuscopeptin production. This is not unlikely given their phylogenetic clustering with LuxR family proteins associated with mycin, peptin and factin BGCs from other *Pseudomonas* species (Girard et al., 2020).

It is often reported that *P. fuscovaginae* can affect panicle formation and grain filling (Batoko et al., 1997; Peeters et al., 2020). In this study, we clearly show the link between CLPs production and panicle suppression, and how *P. fuscovaginae* uses other currently unidentified virulence factors to overcome plant defenses. In the experiments reported here, we detected a reduction in panicle weight caused by *P. fuscovaginae* with two populations appearing; one with compromised tillers and the other with recovered tillers. These symptoms were often not associated with the sheath rot lesion severity. This is also mirrored by the observation that CLPs are not essential to suppress the tiller growth, but are needed to cause sheath rot symptoms. Often, the affected tillers went through total necrosis even when infected with the triple mutants, unable to synthesize any CLPs. A previous study identified possible factors that affected sheath rot lesions (Patel et al., 2014), but it is not known if the same factors might also play a role in the further development of the disease. Nevertheless, considering the higher reduction in the number of formed panicles, the production of CLPs probably provides the strain with an advantage in disease development and it is likely a crucial trait in field conditions.

Understanding the biology of *P. fuscovaginae* is necessary to develop novel control strategies. This is especially important since sheath rot is an understudied emerging disease. Our studies indicate the importance of fuscopeptin and syringotoxin in disease development that is not only limited to sheath rot lesions but extends to the more agronomically significant impact on the panicles. In addition, this work also concludes that the presence of other yet-unknown virulence factors may cooperate in the pathogenicity process.

## Data availability statement

The datasets presented in this study can be found in online repositories. The names of the repository/repositories and accession number(s) can be found below: https://www.ncbi.nlm.nih.gov/, CP100603.

## Author contributions

EF deletion mutants construction, *in planta* and *in vitro* assays, data analysis visualization and interpretation, writing – original draft. MŠ Asplenin deletion mutants construction, review and editing. VL *P. fuscovaginae* plant infection. AM LC-MS/MS analysis. CC-L NRPS domain analyses phylogenesis. BM *in vitro* assays. EP supervision, funding acquisition, project administration, resources. R.D.M. Supervision, funding acquisition, project administration, resources. VV Supervision, funding acquisition, project administration, resources, review and editing. MH Conceptualization, interpretation, supervision, funding acquisition, project administration, resources, review and editing. All authors contributed to the article and approved the submitted version.

## Funding

This work was funded by the Concerted Research Action grant MEMCLiP from Ghent University (GOA-028-19), and the Fonds de la Recherche Scientifique (FNRS) and Research Foundation Flanders (FWO) Excellence of Science grant RhizoCLiP (EOS ID 30650620), www.rhizoclip.be.

## Conflict of interest

The authors declare that the research was conducted in the absence of any commercial or financial relationships that could be construed as a potential conflict of interest.

## Publisher’s note

All claims expressed in this article are solely those of the authors and do not necessarily represent those of their affiliated organizations, or those of the publisher, the editors and the reviewers. Any product that may be evaluated in this article, or claim that may be made by its manufacturer, is not guaranteed or endorsed by the publisher.

## Abbreviations

KB, King’s B medium; BGCs, Biosynthetic gene clusters; ACN, Acetonitrile; FA, Formic acid; CID, Collision induced dissociation; NRPS, Nonribosomal peptide synthetase; PKS, Polyketide synthase; LC-MS/MS, Liquid chromatography with tandem mass spectrometry; NTC, Non-treated control; C domain, Condensation; C_start_ domain, Lipoinitiation; A domain, Adenylation; PCP domain, Peptidyl carrier protein; TE domain, Thioesterase.

## References

[B1] Arp J. Götze S. Mukherji R. Mattern D. J. García-Altares M. Klapper M. . (2018). Synergistic activity of cosecreted natural products from amoebae-associated bacteria. Proc. Natl. Acad. Sci. 115, 3758–3763. doi: 10.1073/pnas.1721790115 29592954 PMC5899472

[B2] Ballio A. Bossa F. Camoni L. Di Giorgio D. Flamand M.-C. Maraite H. . (1996). Structure of fuscopeptins, phytotoxic metabolites of *Pseudomonas fuscovaginae* . FEBS Lett. 381, 213–216. doi: 10.1016/0014-5793(96)00043-9 8601458

[B3] Baré S. Coiro V. M. Scaloni A. Di Nola A. Paci M. Segre A. L. . (1999). Conformations in solution of the fuscopeptins. Eur. J. Biochem. 266, 484–492. doi: 10.1046/j.1432-1327.1999.00883.x 10561589

[B4] Batoko H. Bouharmont J. Kinet J.-M. Marite H. (1997). Involvement of toxins produced by *Pseudomonas fuscovaginae* in aetiology of rice bacterial sheath brown rot. J. Phytopathol. 145, 525–531. doi: 10.1111/j.1439-0434.1997.tb00361.x

[B5] Batoko H. de Kerchove d’Exaerde A. Kinet J.-M. Bouharmont J. Gage R. A. Maraite H. . (1998). Modulation of plant plasma membrane h+-ATPase by phytotoxic lipodepsipeptides produced by the plant pathogen *Pseudomonas fuscovaginae* . Biochim. Biophys. Acta (BBA) - Biomembranes 1372, 216–226. doi: 10.1016/S0005-2736(98)00060-1 9675287

[B6] Bender C. L. Alarcón-Chaidez F. Gross D. C. (1999). *Pseudomonas syringae* phytotoxins: Mode of action, regulation, and biosynthesis by peptide and polyketide synthetases. Microbiol. Mol. Biol. Rev. 63, 266–292. doi: 10.1128/MMBR.63.2.266-292.1999 10357851 PMC98966

[B7] Bigirimana V. de P. Hua G. K. H. Nyamangyoku O. I. Höfte M. (2015). Rice sheath rot: An emerging ubiquitous destructive disease complex. Front. Plant Sci. 6. doi: 10.3389/fpls.2015.01066 PMC4675855 26697031

[B8] Blin K. Shaw S. Steinke K. Villebro R. Ziemert N. Lee S. Y. . (2019). antiSMASH 5.0: updates to the secondary metabolite genome mining pipeline. Nucleic Acids Res. 47, W81–W87. doi: 10.1093/nar/gkz310 31032519 PMC6602434

[B9] Coraiola M. Paletti R. Fiore A. Fogliano V. Serra M. D. (2008). Fuscopeptins, antimicrobial lipodepsipeptides from *Pseudomonas fuscovaginae*, are channel forming peptides active on biological and model membranes. J. Pept. Sci. 14, 496–502. doi: 10.1002/psc.970 18085513

[B10] D’aes J. Kieu N. P. Léclère V. Tokarski C. Olorunleke F. E. De Maeyer K. . (2014). To settle or to move? the interplay between two classes of cyclic lipopeptides in the biocontrol strain *Pseudomonas CMR12a* . Environ. Microbiol. 16, 2282–2300. doi: 10.1111/1462-2920.12462 24673852

[B11] Debois D. Hamze K. Guérineau V. Le Caër J.-P. Holland I. B. Lopes P. . (2008). *In situ* localisation and quantification of surfactins in a *Bacillus subtilis* swarming community by imaging mass spectrometry. PROTEOMICS 8, 3682–3691. doi: 10.1002/pmic.200701025 18709634

[B12] de Bruijn I. de Kock M. J. D. de Waard P. van Beek T. A. Raaijmakers J. M. (2008). Massetolide a biosynthesis in *Pseudomonas fluorescens* . J. Bacteriology 190, 2777–2789. doi: 10.1128/JB.01563-07 PMC2293227 17993540

[B13] de Bruijn I. Raaijmakers J. M. (2009). Diversity and functional analysis of LuxR-type transcriptional regulators of cyclic lipopeptide biosynthesis in *Pseudomonas fluorescens* . Appl. Environ. Microbiol. 75, 4753–4761. doi: 10.1128/AEM.00575-09 19447950 PMC2708414

[B14] Ditta G. Stanfield S. Corbin D. Helinski D. R. (1980). Broad host range DNA cloning system for gram-negative bacteria: construction of a gene bank of. Rhizobium meliloti Proc. Natl. Acad. Sci. U.S.A. 77, 7347–7351. doi: 10.1073/pnas.77.12.7347 7012838 PMC350500

[B15] Duveiller E. Maraite H. (1990). Bacterial sheath rot of wheat caused by *Pseudomonas fuscovaginae* in the highlands of Mexico. Plant Dis. 74, 932–935. doi: 10.1094/PD-74-0932

[B16] Duveiller E. Miyajima K. Snacken F. Autrique A. Maraite H. (1988). Characterization of *Pseudomonas fuscovaginae* and differentiation from other fluorescent pseudomonads occurring on rice in Burundi. J. Phytopathol. 122, 97–107. doi: 10.1111/j.1439-0434.1988.tb00995.x

[B17] Flamand M.-C. Pelsser S. Ewbank E. Maraite H. (1996). Production of syringotoxin and other bioactive peptides by *Pseudomonas fuscovaginae* . Physiol. Mol. Plant Pathol. 48, 217–231. doi: 10.1006/pmpp.1996.0019

[B18] Geudens N. Martins J. C. (2018). Cyclic lipodepsipeptides from pseudomonas spp. – biological Swiss-army knives. Front. Microbiol. 9. doi: 10.3389/fmicb.2018.01867 PMC6104475 30158910

[B19] Geudens N. Nasir M. N. Crowet J. M. Raaijmakers J. M. Fehér K. Coenye T. . (2017). Membrane interactions of natural cyclic lipodepsipeptides of the viscosin group. Biochim. Biophys. Acta - Biomembranes 1859, 331–339. doi: 10.1016/j.bbamem.2016.12.013 28007479

[B20] Girard L. Geudens N. Pauwels B. Höfte M. Martins J. C. Mot R. D. (2022). Transporter gene-mediated typing for detection and genome mining of lipopeptide-producing pseudomonas. Appl. Environ. Microbiol. 88 (2), e01869-21. doi: 10.1128/AEM.01869-21 PMC8788793 34731056

[B21] Girard L. Höfte M. Mot R. D. (2020). Lipopeptide families at the interface between pathogenic and beneficial pseudomonas-plant interactions. Crit. Rev. Microbiol. 46, 397–419. doi: 10.1080/1040841X.2020.1794790 32885723

[B22] Girard L. Lood C. Höfte M. Vandamme P. Rokni-Zadeh H. van Noort V. . (2021). The ever-expanding pseudomonas genus: Description of 43 new species and partition of the *Pseudomonas putida* group. Microorganisms 9, 1766. doi: 10.3390/microorganisms9081766 34442845 PMC8401041

[B23] Gonzalez C. F. DeVay J. E. Wakeman R. J. (1981). Syringotoxin: a phytotoxin unique to citrus isolates of Pseudomonas syringae. Physiological Plant Pathology 18, 41–50. doi: 10.1016/S0048-4059(81)80052-5

[B24] Götze S. Stallforth P. (2020). Structure, properties, and biological functions of nonribosomal lipopeptides from pseudomonads. Natural Product Rep. 37, 29–54. doi: 10.1039/c9np00022d 31436775

[B25] Gross H. Loper J. E. (2009). Genomics of secondary metabolite production by pseudomonas spp. Nat. Prod. Rep. 26, 1408–1446. doi: 10.1039/B817075B 19844639

[B26] Hanahan D. (1985). DNA Cloning: a practical approach (United Kingdom: D. M. Glover).

[B27] Hilgarth R. S. Lanigan T. M. (2020). Optimization of overlap extension PCR for efficient transgene construction. MethodsX 7, 100759. doi: 10.1016/j.mex.2019.12.001 32021819 PMC6992990

[B28] Hmelo L. R. Borlee B. R. Almblad H. Love M. E. Randall T. E. Tseng B. S. . (2015). Precision-engineering the *Pseudomonas aeruginosa* genome with two-step allelic exchange. Nat. Protoc. 10, 1820–1841. doi: 10.1038/nprot.2015.115 26492139 PMC4862005

[B29] Hoang T. T. Karkhoff-Schweizer R. R. Kutchma A. J. Schweizer H. P. (1998). A broad-host-range flp-FRT recombination system for site-specific excision of chromosomally-located DNA sequences: application for isolation of unmarked *Pseudomonas aeruginosa* mutants. Gene 212, 77–86. doi: 10.1016/S0378-1119(98)00130-9 9661666

[B30] Hua G. K. H. Bertier L. Soltaninejad S. Höfte M. (2014). Cropping systems and cultural practices determine the *Rhizoctonia* anastomosis groups associated with *Brassica* spp. in Vietnam. PloS One 9, e111750. doi: 10.1371/journal.pone.0111750 25372406 PMC4221111

[B31] Hunter J. D. (2007). Matplotlib: A 2D graphics environment. Computing Sci. Eng. 9, 90–95. doi: 10.1109/MCSE.2007.55

[B32] Johnson M. Zaretskaya I. Raytselis Y. Merezhuk Y. McGinnis S. Madden T. L. (2008). NCBI BLAST: a better web interface. Nucleic Acids Res. 36, W5–W9. doi: 10.1093/nar/gkn201 18440982 PMC2447716

[B33] Lâm Bạch V. (2021)Pseudomonas and bacillus spp. associated with rice grown in acid sulfate soils in Vietnam. In: Taxonomy and potential for biocontrol and bistimulation. Available at: http://hdl.handle.net/1854/LU-8731991 (Accessed March 3, 2022).

[B34] Minh B. Q. Schmidt H. A. Chernomor O. Schrempf D. Woodhams M. D. von Haeseler A. . (2020). IQ-TREE 2: New models and efficient methods for phylogenetic inference in the genomic era. Mol. Biol. Evol. 37, 1530–1534. doi: 10.1093/molbev/msaa015 32011700 PMC7182206

[B35] Musonerimana S. Bez C. Licastro D. Habarugira G. Bigirimana J. Venturi V. (2020). Pathobiomes revealed that *Pseudomonas fuscovaginae* and *Sarocladium oryzae* are independently associated with rice sheath rot. Microb. Ecol. 80, 627–642. doi: 10.1007/s00248-020-01529-2 32474660

[B36] Nerey Y. Pannecoucque J. Hernandez H. P. Diaz M. Espinosa R. De Vos S. . (2010). *Rhizoctonia* spp. causing root and hypocotyl rot in *Phaseolus vulgaris* in Cuba. J. Phytopathol. 158, 236–243. doi: 10.1111/j.1439-0434.2009.01609.x

[B37] Oni F. E. Geudens N. Omoboye O. O. Bertier L. Hua H. G. K. Adiobo A. . (2019). Fluorescent pseudomonas and cyclic lipopeptide diversity in the rhizosphere of cocoyam (*Xanthosoma sagittifolium*). Environ. Microbiol. 21, 1019–1034. doi: 10.1111/1462-2920.14520 30623562

[B38] Oni F. E. Geudens N. Onyeka J. T. Olorunleke O. F. Salami A.E. Omoboye O. O . (2020). Cyclic lipopeptide‐producing Pseudomonas koreensis group strains dominate the cocoyam rhizosphere of a Pythium root rot suppressive soil contrasting with *P. putida* prominence in conducive soils. Environ. Microbiol. 22, 5137–5155. doi: 10.1111/1462-2920.15127 32524747

[B39] Overbeek R. Olson R. Pusch G. D. Olsen G. J. Davis J. J. Disz T. . (2013). The SEED and the rapid annotation of microbial genomes using subsystems technology (RAST). Nucleic Acids Res. 42, D206–D214. doi: 10.1093/nar/gkt1226 24293654 PMC3965101

[B40] Pannecoucque J. Van Beneden S. Höfte M. (2008). Characterization and pathogenicity of *Rhizoctonia* isolates associated with cauliflower in Belgium. Plant Pathol. 57, 737–746. doi: 10.1111/j.1365-3059.2007.01823.x

[B41] Patel H. K. Matiuzzo M. Bertani I. Bigirimana V. de P. Ash G. J. . (2014). Identification of virulence associated loci in the emerging broad host range plant pathogen *Pseudomonas fuscovaginae* . BMC Microbiol. 14, 274. doi: 10.1186/s12866-014-0274-7 25394860 PMC4237756

[B42] Pearce D. A. Bridge P. D. Hawksworth D. L. (2001). “Species concept in sarocladium, the causal agent of sheath rot in rice and bamboo blight,” in Major fungal diseases of rice: Recent advances. Eds. Sreenivasaprasad S. Johnson R. (Dordrecht: Springer Netherlands), 285–292. doi: 10.1007/978-94-017-2157-8_20

[B43] Peeters K. J. Ameye M. Demeestere K. Audenaert K. Höfte M. (2020). Auxin, abscisic acid and jasmonate are the central players in rice sheath rot caused by *Sarocladium oryzae* and *Pseudomonas fuscovaginae* . Rice 13, 78. doi: 10.1186/s12284-020-00438-9 33242152 PMC7691414

[B44] Quibod I. L. Grande G. Oreiro E. G. Borja F. N. Dossa G. S. Mauleon R. . (2015). Rice-infecting pseudomonas genomes are highly accessorized and harbor multiple putative virulence mechanisms to cause sheath brown rot. PLoS One 10, e0139256. doi: 10.1371/journal.pone.0139256 26422147 PMC4589537

[B45] Raaijmakers J. M. De Bruijn I. Nybroe O. Ongena M. (2010). Natural functions of lipopeptides from bacillus and pseudomonas: more than surfactants and antibiotics. FEMS Microbiol. Rev. 34, 1037–1062. doi: 10.1111/j.1574-6976.2010.00221.x 20412310

[B46] Rott P. Honegger J. Notteghem J. L. Ranomenjanahary S. (1991). Identification of *Pseudomonas fuscovaginae* with biochemical, serological, and pathogenicity tests. Plant Dis. 75, 843–846. doi: 10.1094/PD-75-0843.

[B47] Schindelin J. Arganda-Carreras I. Frise E. Kaynig V. Longair M. Pietzsch T. . (2012). Fiji: an open-source platform for biological-image analysis. Nat. Methods 9, 676–682. doi: 10.1038/nmeth.2019 22743772 PMC3855844

[B48] Serra M. D. Fagiuoli G. Nordera P. Bernhart I. Volpe C. D. Di Giorgio D. . (1999). The interaction of lipodepsipeptide toxins from *Pseudomonas syringae* pv. *syringae* with biological and model membranes: A comparison of syringotoxin, syringomycin, and two syringopeptins. MPMI 12, 391–400. doi: 10.1094/MPMI.1999.12.5.391 10226372

[B49] Simon R. Priefer U. Pühler A. (1983). A broad host range mobilization system for *In vivo* genetic engineering: Transposon mutagenesis in gram negative bacteria. Nat. Biotechnol. 1, 784–791. doi: 10.1038/nbt1183-784

[B50] Sorensen K. N. Kim K. H. Takemoto J. Y. (1996). *In vitro* antifungal and fungicidal activities and erythrocyte toxicities of cyclic lipodepsinonapeptides produced by *Pseudomonas syringae pv. syringae* . Antimicrobial Agents Chemotherapy 40, 2710–2713. doi: 10.1128/AAC.40.12.2710 9124827 PMC163608

[B51] Surico G. DeVay J. E. (1982). Effect of syringomycin and syringotoxin produced by *Pseudomonas syringae pv. syringae* on structure and function of mitochondria isolated from holcus spot resistant and susceptible maize lines. Physiol. Plant Pathol. 21, 39–53. doi: 10.1016/0048-4059(82)90006-6

[B52] The pandas development team (2020). “Pandas-dev/pandas,” in Pandas (Zenodo). doi: 10.5281/zenodo.3509134

[B53] Terpilowski M. (2019). Scikit-posthocs: Pairwise multiple comparison tests in Python. JOSS 4, 1169. doi: 10.21105/joss.01169

[B54] Van Rossum G. Drake F. L. (2009). Python 3 reference manual (Scotts Valley, CA: CreateSpace).

[B55] Virtanen P. Gommers R. Oliphant T. E. Haberland M. Reddy T. Cournapeau D. . (2020). SciPy 1.0: fundamental algorithms for scientific computing in Python. Nat. Methods 17, 261–272. doi: 10.1038/s41592-019-0686-2 32015543 PMC7056644

[B56] Waskom M. L. (2021). Seaborn: statistical data visualization. J. Open Source Software 6, 3021. doi: 10.21105/joss.03021

[B57] Weeraratne N. Stodart B. J. Venturi V. Hofte M. Hua G. K. H. Ongena M. . (2020). Syringopeptin contributes to the virulence of *Pseudomonas fuscovaginae*, based on sypA biosynthesis mutant analysis. Phytopathology® 110, 780–789. doi: 10.1094/PHYTO-07-19-0235-R 31804903

[B58] Ye J. Coulouris G. Zaretskaya I. Cutcutache I. Rozen S. Madden T. L. (2012). Primer-BLAST: A tool to design target-specific primers for polymerase chain reaction. BMC Bioinf. 13, 134. doi: 10.1186/1471-2105-13-134 PMC3412702 22708584

[B59] Zeigler R. S. Alvarez E. (1987). Bacterial sheath brown rot of rice caused by *Pseudomonas fuscovaginae* in Latin America. Plant Dis. 71, 592–597. doi: 10.1094/PD-71-0592

