# Supplementary material for: Versatile role of Pseudomonas fuscovaginae cyclic lipopeptides in plant and microbial interactions
Source: Front Plant Sci. 2022 Nov 8;13:1008980. doi: 10.3389/fpls.2022.1008980 (PMC9679282; doi:10.3389/fpls.2022.1008980)
Supplement: Supplementary file 1 [file DataSheet_1.pdf]

Versatile role of *Pseudomonas fuscovaginae* cyclic lipopeptides in plant and microbial interactions

Enrico Ferrarini, Mihael Špacapan, Van Bach Lam, Andrea McCann; Catherine Cesa-Luna, Bishnu Prasad Marahatta, Edwin De Pauw, René De Mot, Vittorio Venturi, Monica Höfte

Supplementary material

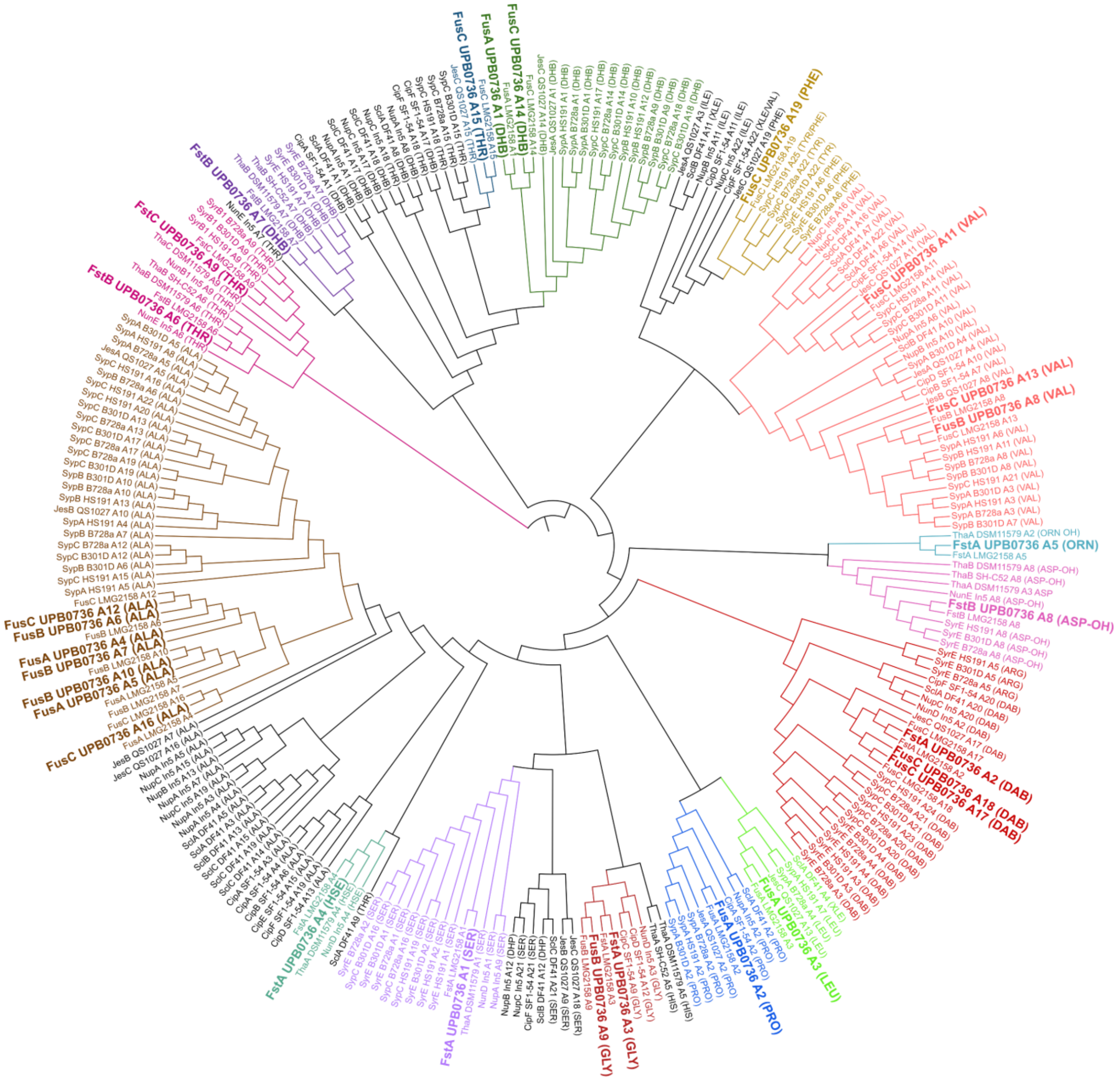

Figure S1: Phylogeny-based substrate prediction of *P. fuscovaginae* UPB0736 fuscopeptin (Fus) and syringotoxin (Fst) synthetases. Cladogram of maximum-likelihood tree inferred from amino

acid sequences alignment of adenylation (A) domains extracted from functionally characterized and putative *Pseudomonas* LP NRPSs. LP-specific codes are indicated as follows: Cip (cichopeptin, *P. cichorii* SF1-54); Fst (putative syringotoxin, *P. fuscovaginae* LMG 2158^T); Fus (putative fuscopeptin, *P. fuscovaginae* LMG 2158^T); Jes (jessenipeptin, *Pseudomonas* sp. QS1027); Nun (nunamycin, *Pseudomonas* sp. In5); Nup (nunapeptin, *Pseudomonas* sp. In5); Scl (sclerosin, *P. brassicacearum* DF41); Syp (syringopeptin, *P. syringae* pv. *syringae* B301D, *P. syringae* pv. *syringae* B728a, *P. syringae* pv. *syringae* HS191); Syr (syringomycin, *P. syringae* pv. *syringae* B301D, *P. syringae* pv. *syringae* B728a, *P. syringae* pv. *syringae* HS191); Tha (thanamycin, *Pseudomonas* sp. DSM 11579, *Pseudomonas* sp. SH-C52). For each domain the substrate specificity (known or inferred) is indicated in parentheses using the standard amino acid three-letter code. HSE: homoserine; DHP: dehydro-2-aminopropanoic acid; XLE: LEU or ILE; DAB: 2,4-diaminobutyric acid; ASP-OH: 3-hydroxy-aspartic acid; ORN-OH: hydroxylation at the α-carbon of ornithine; DHB: 2,3-dehydroaminobutyric acid. Clusters comprising P. *fuscovaginae* UPB0736 A-domains (in bold) are indicated in different colors. See **Table S3** for strain information.

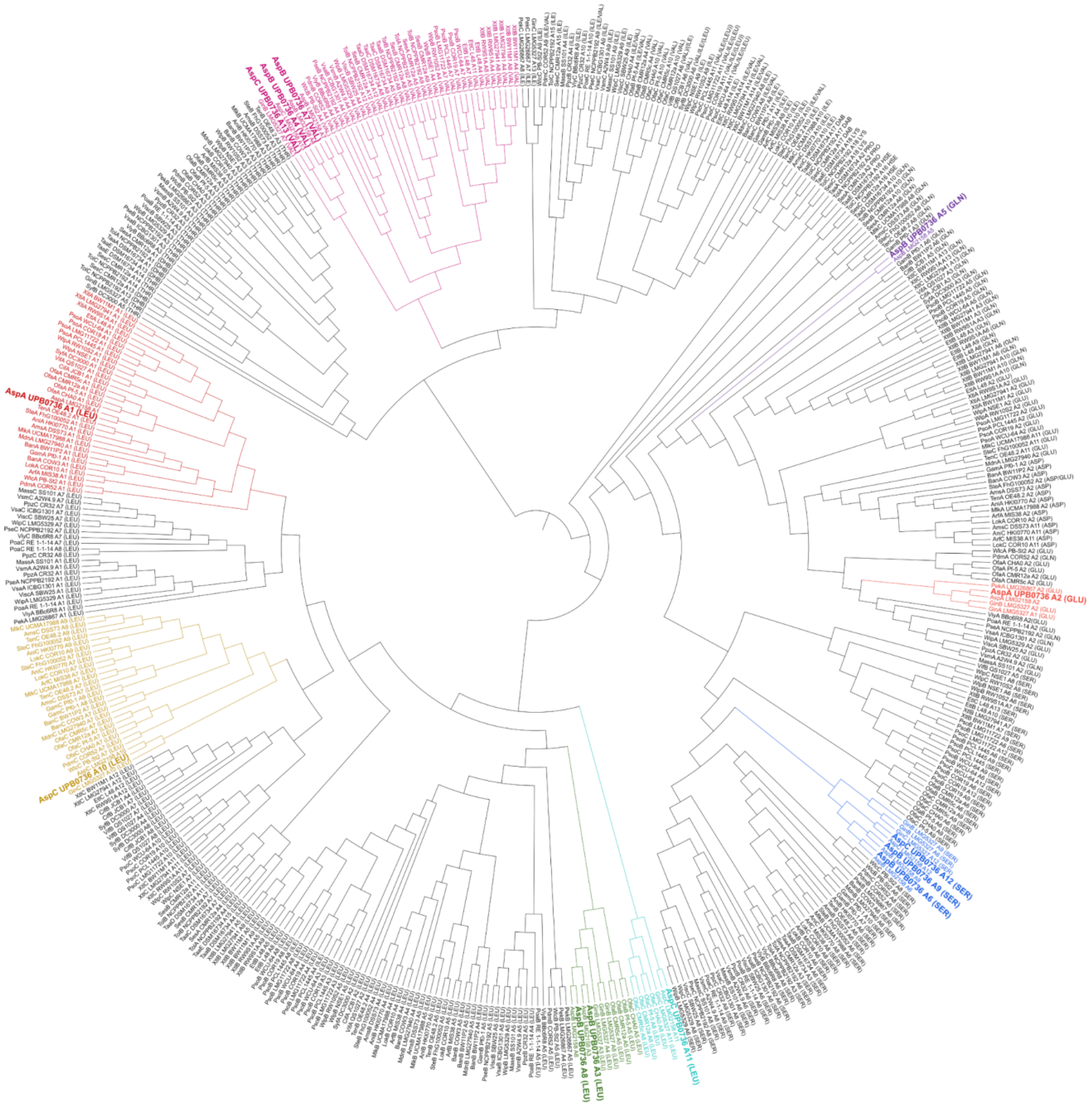

Figure S2. Phylogeny-based substrate prediction of *P. fuscovaginae* UPB0736 asplenin (Asp) synthetases. Cladogram of maximum-likelihood tree inferred from amino acid sequences alignment of adenylation (A) domains extracted from functionally characterized and putative *Pseudomonas* lipopeptide (LP) NRPSs. LP-specific codes are indicated as follows: Ams (amphisin, *P. fluorescens* DSS73); Ani (anikasin, *P. fluorescens* HK10770); Arf (arthrofactin, *Pseudomonas* sp. MIS38); Asp (putative asplenin, *P. fuscovaginae* LMG 2158^{T}); Ban (bananamide, *P. bananamidigenes* BW11P2^{T}, *P. botevensis* COW3^{T}); Cif (cichofactin, *P. cichorii* JBC1); Etl (entolysin, *P. entomophila* L48^{T}); Gam (gacamide, *P. fluorescens* Pf0-1); Gin (gingeramide, *P. gingeri* LMG 5327^{T}); Lok (lokisin, *Pseudomonas* sp. COR10); Mass (massetolide, *P. lactis* SS101); Mdn (MDN-066, *P. granadensis* LMG 27940^{T}); Mlk (milkisin, *P. crudilactis* UCMA 17988^{T}); Ofa (orfamide, *P. aestus* CMR5c, *P. protegens* CHA0^{T}, *P. protegens* Pf-5, *P.*

sessilinigenes CMR12a^T^); Pdm (pseudodesmin, *Pseudomonas* sp. COR52); Pek (prosekin, *P. prosekii* LMG 26867^{T}); Poa (poaeamide A, *P. poae* RE*1-1-14); Ppz (poaeamide B, *P. synxantha* CR32); Pse (pseudodesmin, *P. tolaasii* NCPPB 2192^{T}); Pso (putisolvin, *Pseudomonas* sp. COR19, *P. fulva* LMG 11722^{T}, *P. putida* PCL1445, *P. vlassakiae* WCU 64); Ses (sessilin, *P. sessilinigenes* CMR12a^{T}); Ste (stechlisin, *Pseudomonas* sp. FhG100052); Syf (syringafactin, *P. syringae* pv. *tomato* DC3000); Taa (tolaasin F, *P. costantinii* DSM 16734^{T}); Ten (tensin, *P. zeae* OE48.2); Tol (tolaasin I, *P. tolaasii* NCPPB 2192^{T}); Vif (virginiafactin, *Pseudomonas* sp. QS1027); Visc (viscosin, *P. fluorescens* SBW25); Viy (viscosin, *Pseudomonas* sp. BBc6R8); Vsa (viscosinamide, *Pseudomonas* sp. ICBG1301); Vsm (viscosinamide, *Pseudomonas* sp. A2W4.9); Wip (WLIP, *P. fluorescens* LMG 5329); Wlc (WLIP, *P. chlororaphis* PB-St2); Wlp (WLIP, *Pseudomonas* sp. NSE1, *P. wayambapalatensis* RW10S2); Xtl (xantholysin, *P. mosselii* BW11M1, *P. soli* LMG 27941^{T}, *P. xantholysinigenes* RW9S1A^{T}). For each domain the substrate specificity (known or inferred) is indicated in parentheses using the standard amino acid three-letter code. HSE: homoserine; DHB: 2,3-dehydroaminobutyric acid; DAB: 2,4-diaminobutyric acid. Clusters comprising *P. fuscovaginae* UPB0736 A-domains (in bold) are indicated in different colors. See **Table S4** for strain information.

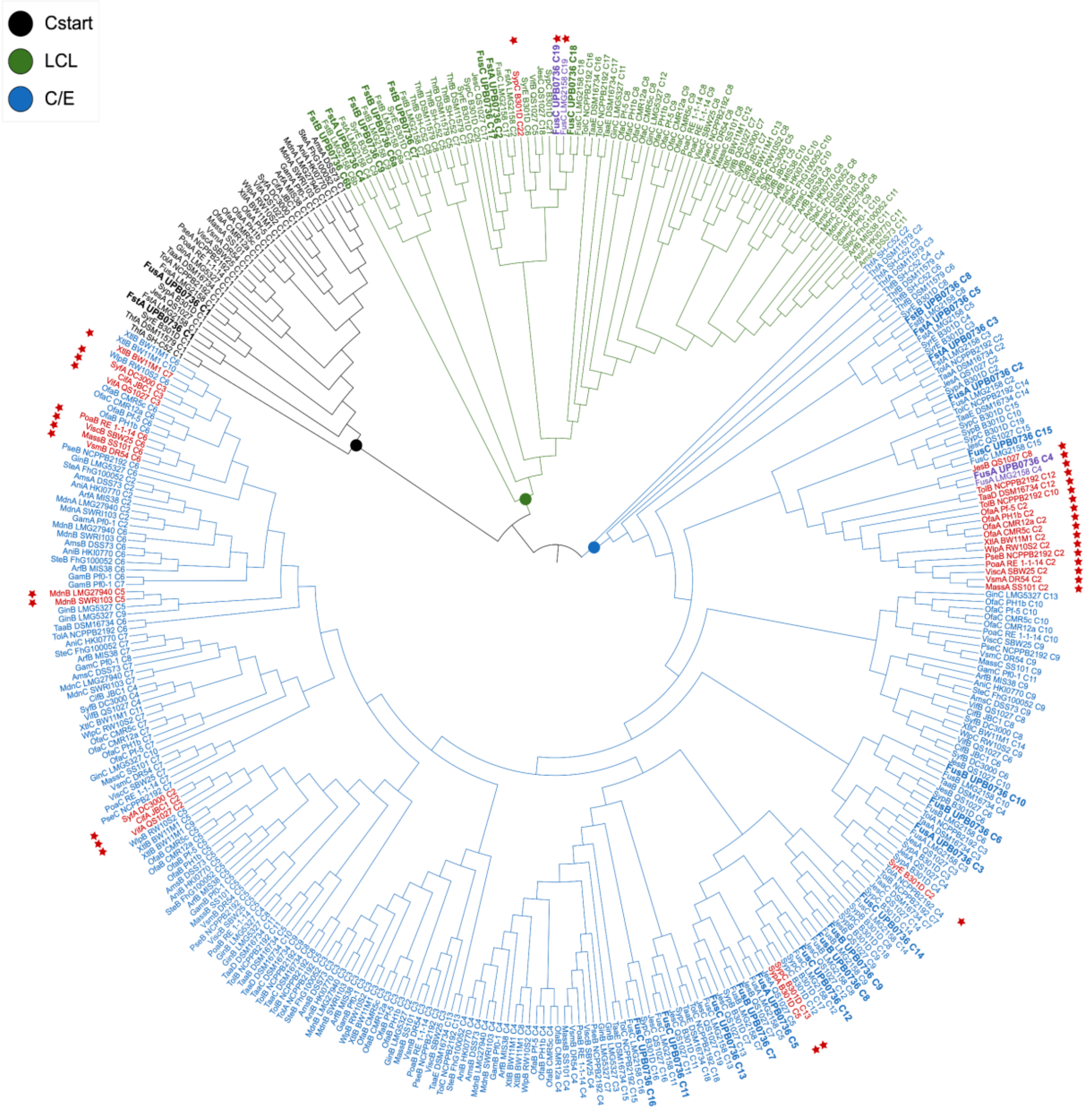

Figure S3. Cladogram of maximum-likelihood tree inferred from amino acid sequence alignment of condensation (C) domains extracted from fuscopeptin (Fus) and syringotoxin (Fst) NRPSs of *P. fuscovaginae* UPB0736 and LMG 2158^{T} , and from C-domains of functionally characterized LP NRPSs from other *Pseudomonas* species. LP-specific codes are indicated as follows: Ams (amphisin, *P. fluorescens* DSS73); Ani (anikasin, *P. fluorescens* HK10770); Arf (arthrofactin, *Pseudomonas* sp. MIS38); Cif (cichofactin, *P. cichorii* JBC1); Gam (gacamide, *P. fluorescens* Pf0-1); Gin (gingeramide, *P. gingeri* LMG 5327^{T}); Mass (massetolide, *P. lactis* SS101); Mdn (MDN-066, *P. azadiae* SWRI103^{T}, *P. granadensis* LMG 27940^{T}); Ofa (orfamide, *P. aestus* CMR5c, *P. protegens* Pf-5, *P. sessilinigenes* CMR12a^{T}, *Pseudomonas* sp. Ph1b); Poa (poaeamide A, *P. poae* RE*1-1-14); Pse (pseudodesmin, *P. tolaasii* NCPPB 2192^{T}); Ste (stechlisin, *Pseudomonas* sp. FhG100052); Syf (syringafactin, *P. syringae* pv. *tomato* DC3000); Taa (tolaasin F, *P. costantinii*

DSM 16734^{T}); Thf (thanafactin, *P. fluorescens* DSM 11579, *Pseudomonas* sp. SH-C52); Tol (tolaasin I, *P. tolaasii* NCPPB 2192^{T}); Vif (virginiafactin, *Pseudomonas* sp. QS1027); Visc (viscosin, *P. fluorescens* SBW25); Vsm (viscosinamide, *Pseudomonas* sp. DR54); Wlp (WLIP, *P. wayambapalatensis* RW10S2); Xtl (xantholysin, *P. mosselii* BW11M1). Non-functional C-domains (in red) and *P. fuscovaginae* C-domains that do not behave as predicted (in purple), are indicated with a red star.

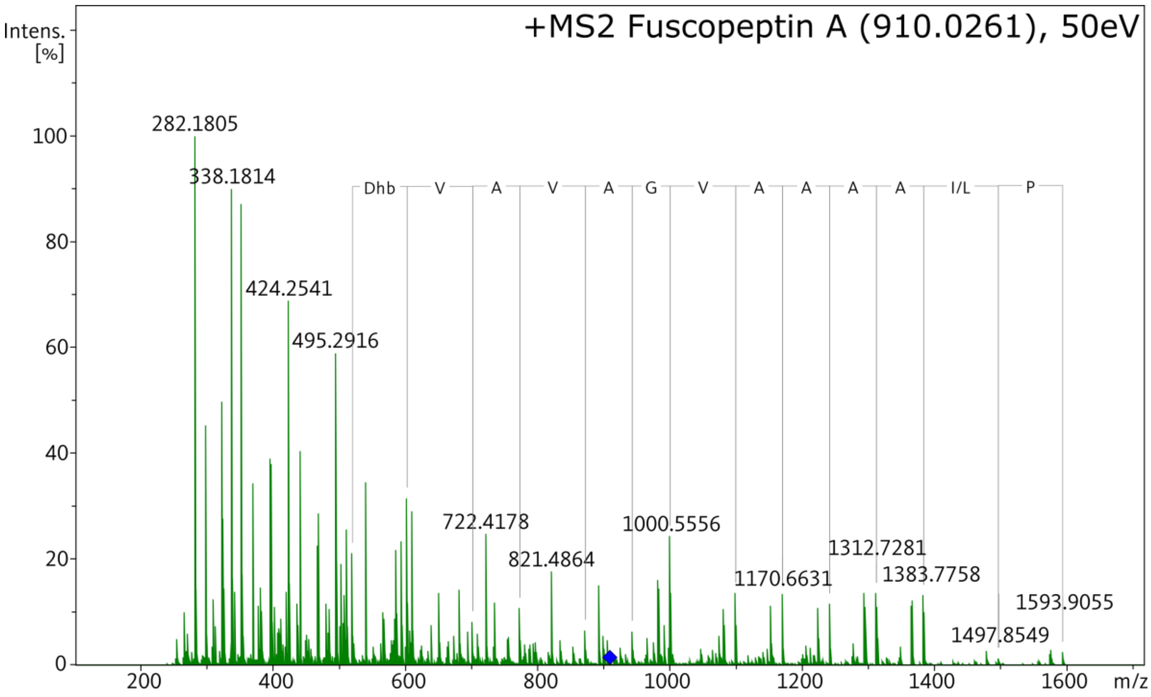

Figure S4: MS/MS spectra of doubly charged fuscopeptin A (M+2H+). Fuscopeptin A was fragmented by Collision Induced Dissociation (CID) with an energy of 50eV.

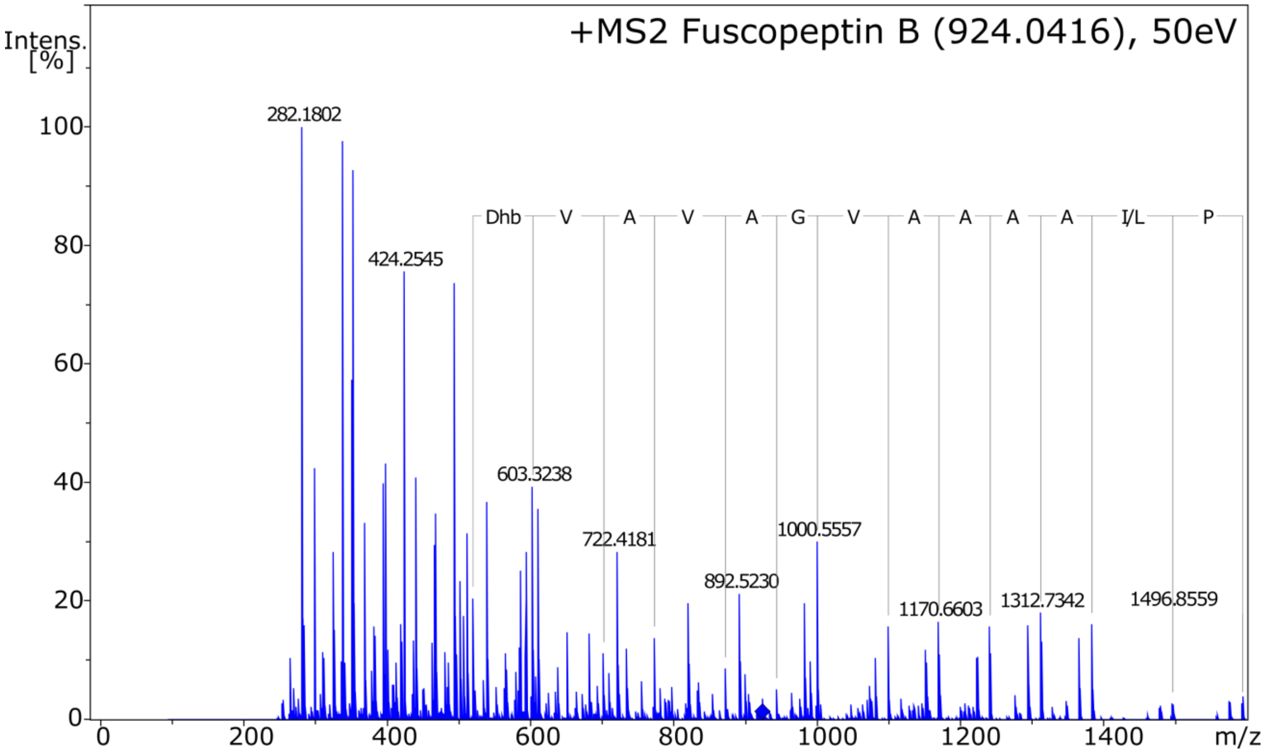

Figure S5: MS/MS spectra of doubly charged fuscopeptin B (M+2H+). Fuscopeptin B was fragmented by CID with an energy of 50eV.

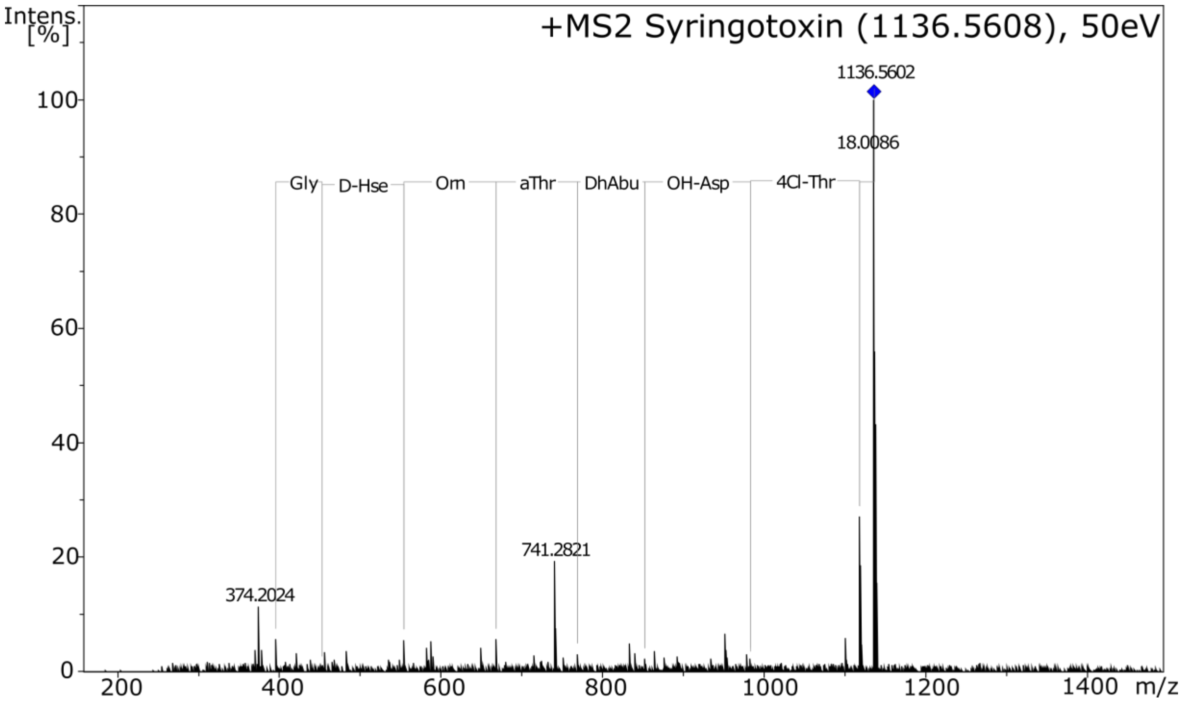

Figure S6: MS/MS spectra of singly charged Syringotoxin ($M+H^+$). Syringotoxin was fragmented by CID with an energy of 50eV.

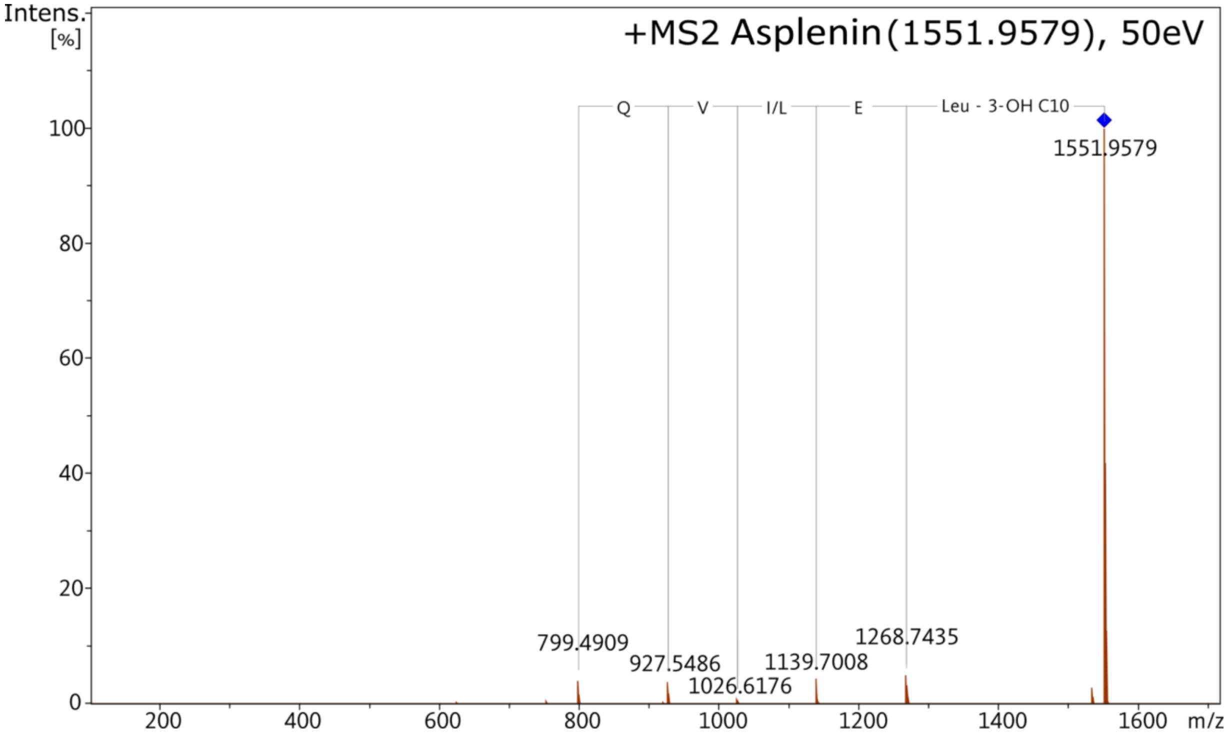

Figure S7: MS/MS spectra of singly charged asplenin ($M+H^+$). Asplenin was fragmented by CID with an energy of 50eV.

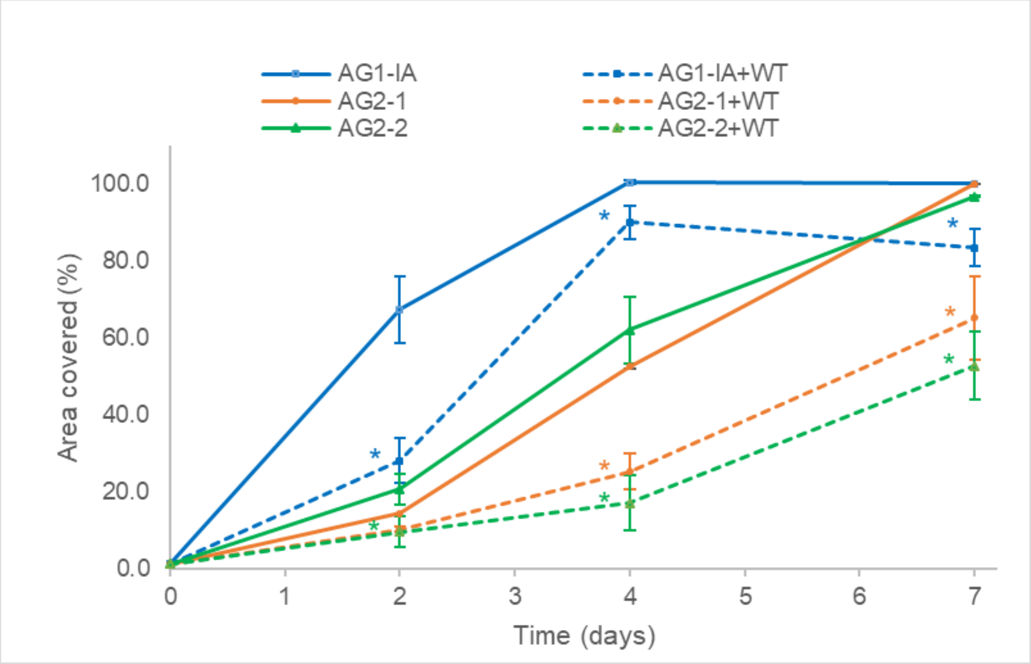

Figure S8. Growth rate of *Rhizoctonia solani* AG1-IA (blue), *R. solani* AG2-1 (orange) and *R. solani* AG2-2 (green) on 1/5 strength PDA in the absence (full line) and presence (dotted line) of *Pseudomonas fuscovaginae* UPB0736 (WT). Ten µl of the *P. fuscovaginae* UPB0736 culture was spotted on two sides of the plate at 2 cm from the central 5 mm agar plug with *R. solani*. Each condition was prepared in triplicates. The plates were cultured at 28 °C and pictures were taken at 2, 4, and 7 days post-inoculation. The mycelial area was measured with Fiji imageJ2 V2.6.0. Data are expressed as percentage of the full plate covered. Vertical bars indicate the standard deviations. Asterisks indicate significant differences compared to the respective *R. solani* control in the absence of *P. fuscovaginae* at the indicated time points.

Table S1. List of primers used for mutant construction in *P. fuscovaginae* UPB0736. Primer sequences in lower case indicate the binding site, upper cases indicate the overhangs.

**Primer name**	**Target gene and overhangs**	**Primer sequence 5'-3'**
UPB_*fst*A_Down_FW	*fstA*, downstream homology, *Eco*RI overhang	CGGAATTCCGatccgtcgggtaatcccgttg
UPB_*fst*A_Down_RV	*fstA*, downstream homology, UPB_*fst*_Up_FW overhang	GCATGTTCGCGATGCTCGAActggataacgagaccgacgc
UPB_*fst*A_Up_FW	*fstA*, upstream homology, UPB_*fst*_Down_RV overhang	GCGTCGGTCTCGTTATCCAGttcgagcatcgcgaacatgc
UPB_*fst*A_Up_RV	*fst*A, upstream homology, *Xba*AI overhang	GCTCTAGAGCgaactggcttcggtccagca
UPB_*fusA*_Up_FW	*fusA*, upstream homology, *Eco*RI overhang	CGGAATTCCGacaacatcggtggctatctgg
UPB_*fusA*_Up_RV	*fusA*, upstream homology, UPB_*fusA*_Down_FW overhang	CAGATAGGGATCGCCCTGTTCtcgaagacttgaacactcggg
UPB_*fusA*_Down_FW	*fusA*, downstream homology, UPB_*fusA*_Up_RV overhang	CCCGAGTGTTCAAGTCTTCGAgaacagggcgatccctatctg
UPB_*fusA*_Down_RV	*fusA*, downstream homology, *Xba*I overhang	GCTCTAGAGCtctgcatgtaaccacagaccc
UPB_aspA_Up_FW	*aspA*, upstream homology, *Eco*RI overhang	CGGAATTCCGagcaaatggcccaggcttat
UPB_*asp*A_Up_RV	*aspA*, upstream homology, UPB_*Asp*A_Down_FW overhang	CGTAAGCTCTCGATCGTCGcgctcatcaggatgactcggg
UPB_*asp*A_Down_FW	*aspA*, downstream homology, UPB_*Asp*A_Up_RV overhang	CCCGAGTCATCCTGATGAGcgacgatcgagagcttacgca
UPB_*asp*A_Down_RV	*aspA*, downstream homology, *Xba*I overhang	GCTCTAGAGcgagcatgctgcgaaagaagg

Table S2. *P. fuscovaginae* UPB0736 WT and mutants LC-MS/MS CLPs quantitation. Samples were taken from KB and LB agar plates. The table show the absolute values of the measured peak areas.

	Genotype			LC-MS/MS peak area (absolute values)							
				Fuscopeptin A		Fuscopeptin B		Syringotoxin		Asplenin	
**Strain**	Fuscopeptins	Syringotoxin	Asplenin	KB	LB	KB	LB	KB	LB	KB	LB
UPB0736-WT	+	+	+	7.02E+06	3.23E+05	1.27E+07	1.37E+06	1.38E+06	2.19E+04	7.81E+06	2.54E+06
UPB0736-Δ*asp*	+	+	-	5.19E+06	1.92E+05	1.14E+07	5.50E+05	9.80E+05	1.10E+04	N/A	N/A
UPB0736-Δ*fst*	+	-	+	5.34E+06	1.02E+05	1.00E+07	3.54E+05	N/A	N/A	4.31E+06	8.82E+05
UPB0736-Δ*fus*	-	+	+	1.10E+03	N/A	7.50E+03	1.44E+03	2.45E+05	1.12E+04	2.61E+06	3.08E+06
UPB0736-Δ*fst*-Δ*fus*	-	-	+	6.53E+03	N/A	2.10E+04	1.73E+03	N/A	N/A	5.23E+06	3.78E+06
UPB0736-Δ*asp*-Δ*fus*	-	+	-	2.89E+03	N/A	1.04E+04	1.24E+03	6.60E+05	2.31E+04	N/A	N/A
UPB0736-Δ*asp*-Δ*fst*	+	-	-	1.10E+06	3.84E+04	3.21E+06	1.23E+05	N/A	N/A	N/A	N/A
UPB0736-Δ*asp*-Δ*fst*-Δ*fus*	-	-	-	3.05E+03	N/A	8.85E+03	9.94E+02	N/A	N/A	N/A	N/A

Table S3 Known Mycin and Peptin-type CLP producers and BGC accession nr.

**Lipopeptide**	**Name**	**Species**	**Strain**	**Accession nr**
**MYCINS**				
Nunamycin	Nun	*Pseudomonas* sp.	In5	LIRD01000002
Syringomycin	Syr	*P. syringae* pv. *syringae*	B301D	CP005969
Syringomycin	Syr	*P. syringae* pv. *syringae*	B728a	CP000075
Syringomycin	Syr	*P. syringae* pv. *syringae*	HS191	CP006256
Syringotoxin (predicted)	Fst	*P. fuscovaginae*	LMG 2158^T	LT629972
Thanamycin	Tha	*Pseudomonas* sp.	DSM 11579	KT362216; JAAOIQ010000001
Thanamycin	Tha	*Pseudomonas* sp.	SH-C52	CBLV010000330
**PEPTINS**				
Cichopeptin	Cip	*P. cichorii*	SF1-54	KJ513094
Fuscopeptin (predicted)	Fus	*P. fuscovaginae*	LMG 2158^T	LT629972
Jessenipeptin	Jes	*Pseudomonas* sp.	QS1027	PHSU00000000
Nunapeptin	Nup	*Pseudomonas* sp.	In5	LIRD01000002
Sclerosin	Scl	*P. brassicacearum*	DF41	CP007410
Syringopeptin SP22	Syp	*P. syringae* pv. *syringae*	B301D	CP005969
Syringopeptin SP22(Phv)	Syp	*P. syringae* pv. *syringae*	B728a	CP000075
Syringopeptin SP25	Syp	*P. syringae* pv. *syringae*	HS191	CP006256

^{T}: type strain

Table S4 Characterized *Pseudomonas* CLPs and BGC accession nr.

Lipopeptide	Name	Species	Strain	Accession nr (BGC unsplit)	Accession nr (BGC split)	
					A-region	BC-region
Amphisin	Ams	*P. fluorescens*	DSS73	JAFLXG010000010		
Anikasin	Ani	*P. fluorescens*	HKI0770	LVEJ01000013		
Arthrofactin	Arf	*Pseudomonas* sp.	MIS38	AB107223		
Asplenin (predicted)	Asp	*P. fuscovaginae*	LMG 2158^T	LT629972		
Bananamide (A-C)	Ban	*P. bananamidigenes*	BW11P2^T	KX437753		
Bananamide (D-G)	Ban	*P. botevensis*	COW3^T	MN480426		
Cichofactin A/B	Cif	*P. cichorii*	JBC1	CP007039		
Entolysin	Etl	*P. entomophila*	L48^T		CT573326	CT573326
Gacamide	Gam	*P. fluorescens*	Pf0-1	CP000094		
Gingeramide (unpublished)	Gin	*P. gingeri*	LMG 5327^T	POWE01000001		
Lokisin	Lok	*Pseudomonas* sp.	COR10	MK534107		
Massetolide	Mass	*P. lactis*	SS101		EU199080	EU199081
MDN-0066	Mdn	*P. granadensis*	LMG 27940^T	LT629778		
		P. azadiae	SWRI103^T	JABBCM010000002		
Milkisin	Mlk	*P. crudilactis*	UCMA 17988^T	WXVV01000014		
Orfamide	Ofa	*P. aestus*	CMR5c	KT613918		
		P. protegens	CHA0^T	CP003190		
		P. protegens	Pf-5	CP000076		
		P. sessilinigenes	CMR12a^T	JQ309921; CP027706		
		Pseudomonas sp.	PH1b	JAAARL010000025		
Pseudodesmin	Pdm	*Pseudomonas* sp.	COR52	MT577358		
Prosekin	Pek	*P. prosekii*	LMG 26867^T	LT629762		
Poaeamide A	Poa	*P. poae*	RE*1-1-14		CP004045	CP004045
Poaeamide B	Ppz	*P. synxantha*	CR32		KU936045	KU936046
Pseudodesmin	Pse	*P. tolaasii*	NCPPB 2192^T = LMG2342		PHHD0100 0001	PHHD0100 0001
Putisolvin	Pso	*Pseudomonas* sp.	COR19	MT511055		
		P. putida	PCL1445	DQ151887		
		P. fulva	LMG 11722^T	BBIQ01000007		
		P. vlassakiae	WCU 64	MT511054		
Sessilin	Ses	*P. sessilinigenes*	CMR12a^T	CP027706		
Stechlisin/tensin	Ste	*Pseudomonas* sp.	FhG1000052	MT080808		
Syringafactin	Syf	*P. syringae pv. tomato*	DC3000	AE016853		
Tolaasin F	Taa	*P. costantinii*	DSM 16734^T = LMG22119^T	HE967327		

Tensin	Ten	*P. zeae*	OE 48.2[T]	CP077090		
Thanafactin	Thf	*P. fluorescens*	DSM11579	JAAOIQ010000001		
		Pseudomonas sp.	SH-C52	MT431590		
Tolaasin I/II/B/D/E	Tol	*P. tolaasii*	NCPPB 2192[T] = LMG2342	PHHD01000001		
Virginiafactin A/B/C	Vif	*Pseudomonas* sp.	QS1027	PHSU01000000		
Viscosin	Visc	*P. fluorescens*	SBW25		AM181176	AM181176
	Viy	*Pseudomonas* sp.	BBc6R8	AKXH02000048 & AKXH02000047		
Viscosinamide	Vsa	*Pseudomonas* sp.	ICBG1301		JAEKGB01<br>0000001	JAEKGB01<br>0000009
	Vsm	*Pseudomonas* sp.	A2W4.9		MT749674	MT771985
		P. carnis	DR54		JAFLXE01<br>0000039	JAFLXE01<br>0000037
WLIP	Wip	*P. fluorescens*	LMG 5329		JQ974025	JQ974026
	Wlc	*P. chlororaphis*	PB-St2	CP027716		
	Wlp	*P. wayambapalatensis*	RW10S2		JN982332	JN982333
	Wlp	*Pseudomonas* sp.	NSE1		MK534106	MK650230
Xantholysin	Xtl	*P. mosselii*	BW11M1	KC297505 & LSLE01000008	KC297506	
		P. soli	LMG 27941[T]	FOEQ01000005		
		P. xantholysinigenes	RW9S1A[T]	CP077095	CP077095	

[T]: type strain

Table S5. P-values for brown sheath rot lesion length (Fig 4b). Rice plants were inoculated one week before booting stage with *P. fuscovaginae* UPB0736 or one of its CLP mutants. P-values were calculated after Kruskal-Wallis test with a post hoc pairwise Dunn tests with Holm-Sidak correction. $P \leq 5.00e\text{-}02$ are highlighted in red.

**Strain**	NTC	WT	*Δasp*	*Δfst*	*Δfus*	*Δfst-Δfus*	*Δasp-Δfus*	*Δasp-Δfst*	*Δasp-Δfst-Δfus*
NTC		0.00E+00	3.00E-06	0.00E+00	6.45E-02	1.00E+00	6.17E-03	0.00E+00	1.00E+00
WT	0.00E+00		4.83E-01	8.50E-01	3.97E-04	0.00E+00	7.71E-03	8.92E-01	0.00E+00
Δasp	3.00E-06	4.83E-01		9.84E-01	2.00E-01	3.00E-06	6.44E-01	9.84E-01	3.00E-06
Δfst	0.00E+00	8.50E-01	9.84E-01		4.81E-02	0.00E+00	2.64E-01	1.00E+00	0.00E+00
Δfus	6.45E-02	3.97E-04	2.00E-01	4.81E-02		6.45E-02	9.84E-01	3.16E-02	6.45E-02
Δfst-Δfus	1.00E+00	0.00E+00	3.00E-06	0.00E+00	6.45E-02		6.17E-03	0.00E+00	1.00E+00
Δasp-Δfus	6.17E-03	7.71E-03	6.44E-01	2.64E-01	9.84E-01	6.17E-03		2.02E-01	6.17E-03
Δasp-Δfst	0.00E+00	8.92E-01	9.84E-01	1.00E+00	3.16E-02	0.00E+00	2.02E-01		0.00E+00
Δasp-Δfst-Δfus	1.00E+00	0.00E+00	3.00E-06	0.00E+00	6.45E-02	1.00E+00	6.17E-03	0.00E+00	

Table S6. P-values for brown sheath rot lesion length (Fig 4c). Rice plants were inoculated two weeks before booting stage with *P. fuscovaginae* UPB0736 or one of its CLP mutants. P-values were calculated after Kruskal-Wallis test with a post hoc pairwise Dunn tests with Holm-Sidak correction. $P \leq 5.00e\text{-}02$ are highlighted in red.

**Strain**	NTC	WT	*Δasp*	*Δfst*	*Δfus*	*Δfst-Δfus*	*Δasp-Δfus*	*Δasp-Δfst*	*Δasp-Δfst-Δfus*
NTC		0.00E+00	1.00E-06	0.00E+00	3.61E-03	1.00E+00	3.41E-02	0.00E+00	1.00E+00
WT	0.00E+00		9.00E-01	9.90E-01	4.29E-02	0.00E+00	5.05E-03	9.83E-01	0.00E+00
Δasp	1.00E-06	9.00E-01		9.97E-01	6.02E-01	1.00E-06	2.04E-01	9.98E-01	1.00E-06
Δfst	0.00E+00	9.90E-01	9.97E-01		2.85E-01	0.00E+00	5.97E-02	1.00E+00	0.00E+00
Δfus	3.61E-03	4.29E-02	6.02E-01	2.85E-01		3.61E-03	9.93E-01	3.50E-01	3.61E-03
Δfst-Δfus	1.00E+00	0.00E+00	1.00E-06	0.00E+00	3.61E-03		3.41E-02	0.00E+00	1.00E+00
Δasp-Δfus	3.41E-02	5.05E-03	2.04E-01	5.97E-02	9.93E-01	3.41E-02		8.28E-02	3.41E-02
Δasp-Δfst	0.00E+00	9.83E-01	9.98E-01	1.00E+00	3.50E-01	0.00E+00	8.28E-02		0.00E+00
Δasp-Δfst-Δfus	1.00E+00	0.00E+00	1.00E-06	0.00E+00	3.61E-03	1.00E+00	3.41E-02	0.00E+00	

Table S7. P-values for brown sheath rot lesion type (Fig 4d). Rice plants were inoculated one week before booting stage with *P. fuscovaginae* UPB0736 or one of the mutants in the CLPs BGCs. P-values were calculated after Kruskal-Wallis test with a post hoc pairwise Dunn tests with Holm-Sidak correction. $P \leq 5.00e\text{-}02$ are highlighted in red.

**Strain**	NTC	WT	*Δasp*	*Δfst*	*Δfus*	*Δfst-Δfus*	*Δasp-Δfus*	*Δasp-Δfst*	*Δasp-Δfst-Δfus*
NTC		0.00E+00	5.00E-06	7.00E-06	1.52E-02	1.00E+00	1.58E-03	0.00E+00	1.00E+00
WT	0.00E+00		6.00E-01	5.70E-01	4.36E-03	0.00E+00	3.42E-02	8.73E-01	0.00E+00
Δasp	5.00E-06	6.00E-01		1.00E+00	5.77E-01	5.00E-06	8.78E-01	9.95E-01	5.00E-06
Δfst	7.00E-06	5.70E-01	1.00E+00		6.01E-01	7.00E-06	8.95E-01	9.95E-01	7.00E-06
Δfus	1.52E-02	4.36E-03	5.77E-01	6.01E-01		1.52E-02	9.95E-01	2.39E-01	1.52E-02
Δfst-Δfus	1.00E+00	0.00E+00	5.00E-06	7.00E-06	1.52E-02		1.58E-03	0.00E+00	1.00E+00
Δasp-Δfus	1.58E-03	3.42E-02	8.78E-01	8.95E-01	9.95E-01	1.58E-03		6.01E-01	1.58E-03
Δasp-Δfst	0.00E+00	8.73E-01	9.95E-01	9.95E-01	2.39E-01	0.00E+00	6.01E-01		0.00E+00
Δasp-Δfst-Δfus	1.00E+00	0.00E+00	5.00E-06	7.00E-06	1.52E-02	1.00E+00	1.58E-03	0.00E+00	

Table S8. P-values for brown sheath rot lesion type (Fig. 4e). Rice plants were inoculated two weeks before booting stage with *P. fuscovaginae* UPB0736 or one of the mutants in the CLPs BGCs. P-values were calculated after Kruskal-Wallis test with a post hoc pairwise Dunn tests with Holm-Sidak correction. $P \leq 5.00e\text{-}02$ are highlighted in red.

**Strain**	NTC	WT	*Δasp*	*Δfst*	*Δfus*	*Δfst-Δfus*	*Δasp-Δfus*	*Δasp-Δfst*	*Δasp-Δfst-Δfus*
NTC		0.00E+00	0.00E+00	1.00E-06	3.13E-03	1.00E+00	2.58E-02	0.00E+00	1.00E+00
WT	0.00E+00		9.93E-01	8.53E-01	4.63E-02	0.00E+00	6.61E-03	9.93E-01	0.00E+00
Δasp	0.00E+00	9.93E-01		9.95E-01	2.96E-01	0.00E+00	7.47E-02	1.00E+00	0.00E+00
Δfst	1.00E-06	8.53E-01	9.95E-01		6.96E-01	1.00E-06	2.90E-01	9.95E-01	1.00E-06
Δfus	3.13E-03	4.63E-02	2.96E-01	6.96E-01		3.13E-03	9.95E-01	2.90E-01	3.13E-03
Δfst-Δfus	1.00E+00	0.00E+00	0.00E+00	1.00E-06	3.13E-03		2.58E-02	0.00E+00	1.00E+00
Δasp-Δfus	2.58E-02	6.61E-03	7.47E-02	2.90E-01	9.95E-01	2.58E-02		6.46E-02	2.58E-02
Δasp-Δfst	0.00E+00	9.93E-01	1.00E+00	9.95E-01	2.90E-01	0.00E+00	6.46E-02		0.00E+00
Δasp-Δfst-Δfus	1.00E+00	0.00E+00	0.00E+00	1.00E-06	3.13E-03	1.00E+00	2.58E-02	0.00E+00	

Table S9. P-values for the rice plants tiller length (Fig 5a). Rice plants were inoculated one week before booting stage with *P. fuscovaginae* UPB0736 or one of the mutants in the CLPs BGCs. P-values were calculated after Kruskal-Wallis test with a post hoc pairwise Dunn tests with Holm-Sidak correction. *P ≤ 5.00e-02* are highlighted in red.

**Strain**	NTC	WT	*Δasp*	*Δfst*	*Δfus*	*Δfst-Δfus*	*Δasp-Δfus*	*Δasp-Δfst*	*Δasp-Δfst-Δfus*
NTC		0.00E+00	1.00E-06	1.60E-05	2.00E-06	2.34E-01	1.00E-06	3.00E-06	1.44E-01
WT	0.00E+00		9.99E-01	9.56E-01	9.98E-01	2.53E-03	9.99E-01	9.96E-01	6.30E-03
Δasp	1.00E-06	9.99E-01		1.00E+00	1.00E+00	5.40E-02	1.00E+00	1.00E+00	9.64E-02
Δfst	1.60E-05	9.56E-01	1.00E+00		1.00E+00	1.70E-01	1.00E+00	1.00E+00	2.76E-01
Δfus	2.00E-06	9.98E-01	1.00E+00	1.00E+00		6.22E-02	1.00E+00	1.00E+00	1.09E-01
Δfst-Δfus	2.34E-01	2.53E-03	5.40E-02	1.70E-01	6.22E-02		4.21E-02	8.23E-02	1.00E+00
Δasp-Δfus	1.00E-06	9.99E-01	1.00E+00	1.00E+00	1.00E+00	4.21E-02		1.00E+00	8.06E-02
Δasp-Δfst	3.00E-06	9.96E-01	1.00E+00	1.00E+00	1.00E+00	8.23E-02	1.00E+00		1.44E-01
Δasp-Δfst-Δfus	1.44E-01	6.30E-03	9.64E-02	2.76E-01	1.09E-01	1.00E+00	8.06E-02	1.44E-01	

Table S10. P-values for the rice plants tiller length (Fig 5b). Rice plants were inoculated two weeks before booting stage with *P. fuscovaginae* UPB0736 or one of the mutants in the CLPs BGCs. P-values were calculated after Kruskal-Wallis test with a post hoc pairwise Dunn tests with Holm-Sidak correction. *P ≤ 5.00e-02* are highlighted in red.

**Strain**	NTC	WT	*Δasp*	*Δfst*	*Δfus*	*Δfst-Δfus*	*Δasp-Δfus*	*Δasp-Δfst*	*Δasp-Δfst-Δfus*
NTC		5.02E-04	5.42E-04	8.10E-04	5.88E-01	1.00E+00	1.67E-01	1.49E-03	3.72E-01
WT	5.02E-04		1.00E+00	1.00E+00	4.38E-01	2.87E-03	8.04E-01	1.00E+00	6.42E-01
Δasp	5.42E-04	1.00E+00		1.00E+00	4.38E-01	3.06E-03	8.04E-01	1.00E+00	6.42E-01
Δfst	8.10E-04	1.00E+00	1.00E+00		5.07E-01	4.38E-03	8.18E-01	1.00E+00	6.85E-01
Δfus	5.88E-01	4.38E-01	4.38E-01	5.07E-01		8.04E-01	9.99E-01	6.06E-01	1.00E+00
Δfst-Δfus	1.00E+00	2.87E-03	3.06E-03	4.38E-03	8.04E-01		4.38E-01	7.53E-03	6.42E-01
Δasp-Δfus	1.67E-01	8.04E-01	8.04E-01	8.18E-01	9.99E-01	4.38E-01		8.78E-01	1.00E+00
Δasp-Δfst	1.49E-03	1.00E+00	1.00E+00	1.00E+00	6.06E-01	7.53E-03	8.78E-01		7.79E-01
Δasp-Δfst-Δfus	3.72E-01	6.42E-01	6.42E-01	6.85E-01	1.00E+00	6.42E-01	1.00E+00	7.79E-01	

Table S11 P-values for the number of emitted panicles (Fig 5c). Rice plants were inoculated one week before booting stage with *P. fuscovaginae* UPB0736 or one of the mutants in the CLPs BGCs. P-values were calculated via post-hoc chi-square tests, Benjamini-Hochberg FDR corrected. Pairwise comparisons with both values at zero were excluded from the analysis. *P ≤ 5.00e-02* are highlighted in red.

**Strain**	WT	*Δasp*	*Δasp-Δfst*	*Δasp-Δfst-Δfus*	*Δasp-Δfus*	*Δfst*	*Δfst-Δfus*	*Δfus*
NTC	1.00E-06	0.00E+00	0.00E+00	4.06E-03	3.00E-06	1.00E-06	7.26E-03	3.00E-06
WT		1.00E+00	1.00E+00	4.58E-02	1.00E+00	1.00E+00	2.30E-02	1.00E+00
Δasp				1.46E-02	6.81E-01	1.00E+00	7.26E-03	6.81E-01
Δasp-Δfst				1.46E-02	6.81E-01	1.00E+00	7.26E-03	6.81E-01
Δasp-Δfst-Δfus					1.10E-01	4.58E-02	1.00E+00	1.10E-01
Δasp-Δfus						1.00E+00	5.82E-02	1.00E+00
Δfst							2.30E-02	1.00E+00
Δfst-Δfus								5.82E-02

Table S12. P-values for the number of emitted panicles (Fig 5d). Rice plants were inoculated two weeks before booting stage with *P. fuscovaginae* UPB0736 or one of the mutants in the CLPs BGCs. P-values were calculated via post-hoc chi-square tests, Benjamini-Hochberg FDR corrected. Pairwise comparisons with both values at zero were excluded from the analysis. *P ≤ 5.00e-02* are highlighted in red.

**Strain**	WT	*Δasp*	*Δasp-Δfst*	*Δasp-Δfst-Δfus*	*Δasp-Δfus*	*Δfst*	*Δfst-Δfus*	*Δfus*
NTC	5.87E-04	3.48E-04	1.54E-04	3.66E-02	3.66E-02	1.54E-04	1.00E+00	1.95E-02
WT		1.00E+00	8.87E-01	2.54E-01	2.54E-01	8.87E-01	2.28E-03	4.23E-01
Δasp				1.49E-01	1.49E-01	1.00E+00	8.47E-04	2.54E-01
Δasp-Δfst				8.75E-02	8.75E-02	1.00E+00	3.48E-04	1.49E-01
Δasp-Δfst-Δfus					1.00E+00	8.75E-02	8.75E-02	1.00E+00
Δasp-Δfus						8.75E-02	8.75E-02	1.00E+00
Δfst							3.48E-04	1.49E-01
Δfst-Δfus								5.87E-04
